# Removal of anthracycline cytostatics from aquatic environment: Comparison of nanocrystalline titanium dioxide and decontamination agents

**DOI:** 10.1371/journal.pone.0223117

**Published:** 2019-10-11

**Authors:** Martin Šťastný, Václav Štengl, Irena Štenglová-Netíková, Michaela Šrámová-Slušná, Pavel Janoš

**Affiliations:** 1 Institute of Inorganic Chemistry of the Czech Academy of Sciences, Řež, Czech Republic; 2 1st Faculty of Medicine, Charles University in Prague, Ovocný trh, Czech Republic; 3 Faculty of the Environment, J.E.Purkyně University in Ústí nad Labem, Ústí nad Labem, Czech Republic; Institute of Materials Science, GERMANY

## Abstract

Anthracyclines are a class of pharmaceuticals used in cancer treatment have the potential to negatively impact the environment. To study the possibilities of anthracyclines (represented by pirarubicin and valrubicin) removal, chemical inactivation using NaOH (0.01 M) and NaClO (5%) as decontamination agents and adsorption to powdered nanocrystalline titanium dioxide (TiO_2_) were compared. The titanium dioxide (TiO_2_) nanoparticles were prepared *via* homogeneous precipitation of an aqueous solution of titanium (IV) oxy-sulfate (TiOSO_4_) at different amount (5–120 g) with urea. The as-prepared TiO_2_ samples were characterized by XRD, HRSEM and nitrogen physisorption. The adsorption process of anthracycline cytostatics was determined followed by high-performance liquid chromatography coupled with mass spectrometry (LC-MS) and an *in-situ* Diffuse Reflectance Infrared Fourier Transform Spectroscopy (DRIFTS) technique. It was found that NaClO decomposes anthracyclines to form various transformation products (TPs). No TPs were identified after the reaction of valrubicin with a NaOH solution as well as in the presence of TiO_2_ nanoparticles. The best degree of removal, 100% of pirarubicin and 85% of valrubicin, has been achieved in a sample with 120 grams of TiOSO_4_ (TIT120) and TiO_2_ with 60 grams (TIT60), respectively.

## 1. Introduction

Due to the dangerous properties of cytostatics and their metabolites, these substances can pose serious health and environmental risks to the environment and human health [1]. Particular attention is, therefore, paid to the presence of residues of these substances in wastewater, leaving medical facilities (hospitals), where these substances are handled [2]. In addition, the biodegradability of these substances in the aquatic environment is very slow. Biologically active compounds thus often pass through municipal wastewater treatment plants unchanged. Wastewater cytotoxicity is also increased when active cytostatic metabolites are excreted [3]. An effective way of wastewater treatment from organic micro-pollutants can be achieved by using adsorption on a variety of nanomaterials including metal nanostructures [4], nanocarbons [5], graphene nanosheets [6,7] or hybrid composites [8] have been explored for the effective removal of organic pollutants. These materials are characterized by high surface area, significant porosity, small particle size, and mechanical strength, as well as the occurrence of a significant number of defects in the crystal lattice and surface functional groups [9]. In the available literature, we can most often encounter nanostructured magnetite (Fe_3_O_4_) adsorbents with a different surface modification for the sorption of platinum cytostatics [10] or nanostructured titanium dioxide (TiO_2_) in combination with the UV/Vis photocatalytic degradation of anthracycline glycoside-based cytostatics [11] and derivates of pyrimidine (5-fluorouracil) [12].

The mechanism of action of these drugs is based on the inhibition of proliferation (multiplication) of rapidly dividing tumor cells. It is, therefore, a drug which non-selectively blocks the growth of tumor cells but, unfortunately, affects the healthy cells too. This might affect the cells of health service workers who handle and are thus exposed to harmful cytostatic drugs, whose effects may be either local (allergic eczema) or generalized (cancer or genetic mutations) [13–15]. Methods of decontamination of the cytostatics are frequently discussed areas of occupational health services that come into contact with these drugs [16]. Some studies that have focused on evaluating the effectiveness of chemical agents then showed that, for example, the commonly used sodium hypochlorite solution (5%) may effectively decompose some cytostatics (but the transformation products of the reactions and their toxicity were not observed) [17,18]. Lamerie *et al*. (2013), has dealt with testing the effectiveness of decontamination agents (e.g., isopropyl alcohol, acetone, sodium hypochlorite, and surfactants) for removal of antineoplastic drugs (e.g., cyclophosphamide, ifosfamide, doxorubicin, or epirubicin). The residual contamination was quantified using high-performance liquid chromatography coupled with mass spectrometry (HPLC-MS). To compare all tested cleaning agents, a sodium hypochlorite showed the highest overall effectiveness, with 98% contamination removed [19]. The current practice uses the chemical conversion of cytotoxic waste into non-toxic residues in the alkaline hydrolysis, chemical oxidation with potassium permanganate or sulfuric acid [20], denitrosation [21], or other effective methods for the particular type of cytotoxic waste [22].

Recently, we have dealt with a detailed study of the degradation of cytostatics pertaining to the class of anthracycline derivatives (doxorubicin and epirubicin) [23], anthraquinone derivates (mitoxantrone) [24] and nitrogen mustards family (cyclophosphamide and ifosfamide) [25] in decontamination agents (NaOH, NaClO) and on the surface of nanostructured adsorbents (TiO_2_, MnO_2_).

**Fig 1:**
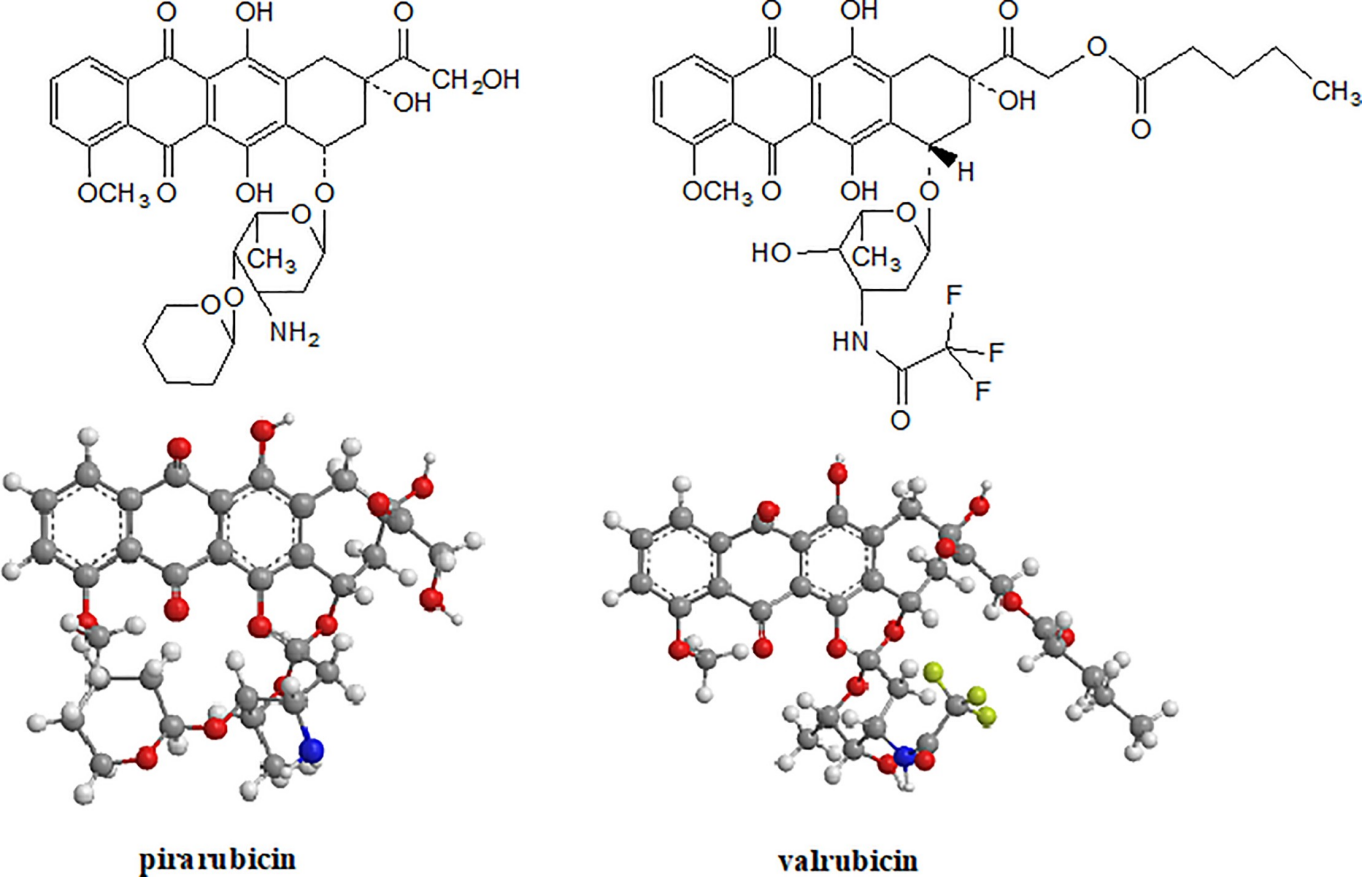
Chemical structures and 3D models of pirarubicin a valrubicin pertaining to the group of anthracycline glycosides.

In this study, we compared the chemical agents (NaClO and NaOH) and the adsorptive method based on nanostructured titanium dioxide (TiO_2_) for the removal of cytostatic drugs of the class of anthracycline derivatives (pirarubicin and valrubicin). Pirarubicin and valrubicin (see Fig 1) are anthracycline derivates of doxorubicin, which are used for intravesical chemotherapy to treat superficial bladder cancer and other solid tumors [26,27]. To investigate the degradation of these cytostatics in sodium hydroxide (0.01 M), sodium hypochlorite (5%) as decontamination agents and on the surface of nanocrystalline TiO_2_ we used the high-performance liquid chromatographic method coupled with mass spectrometry (HPLC-MS) and an *in-situ* Diffuse Reflectance Infrared Fourier Transform Spectroscopy (DRIFTS) technique.

## 2. Experimental

### 2.1. Materials and chemicals

All of the chemicals used, including the titanium oxo-sulfate (TiOSO_4_), urea (CO(NH_2_)_2_), sodium hydroxide (NaOH), sodium hypochlorite (NaClO), sulfuric acid, and PIRA and VAL were obtained from Sigma-Aldrich (St Louis, MO, USA). All the reagents used were of analytical grade and were employed without any further purification. HPLC-grade organic solvents and deionized water were used to prepare the solutions, including mobile phases for liquid chromatography. Lichrosolv® HPLC grade methanol (MeOH) and formic acid (FA) came from Biosolve (Valkenswaard, the Netherlands).

### 2.2. Preparation of titanium dioxide nanoparticles

Titanium dioxide nanoparticles were prepared *via* homogeneous precipitation with urea, which has been described in detail several times [28–30]. In this study, a total of six titanium dioxide samples were prepared, different in the amount of titanium (IV) oxy-sulfate [TiOSO_4_] (5–120 g), as shown in Table 1. In a typical synthesis, given quantity of TiOSO_4_ (see Table 1) was dissolved in 100 mL of hot distilled water acidified with 98% H_2_SO_4_. The pellucid liquid was diluted into 4 L of distilled water and added 300 g of urea. The mixture was heated at 98°C under stirring for 6 h until pH reached 7.2. The formed precipitate was washed using decantation until conductivity of 10μS was reached, filtered off, and dried at 105°C.

**Table 1:** Specific surface area, total pore volume, and the crystallite size of TiO_2_ samples prepared by homogeneous precipitation.

Sample	TiOSO_4_ [g]	Surface area [m^2^·g^-1^]	Total pore volume [cm^3^·g^-1^]	Crystallite size [nm]
TIT5	5	305	0.15	4.2
TIT15	15	319	0.09	4.0
TIT30	30	333	0.16	3.8
TIT60	60	337	0.12	3.6
TIT100	100	340	0.13	3.3
TIT120	120	346	0.06	3.0

### 2.3. Preparation of stock solutions

For the stability testing and degradation studies, stock solutions of drugs in deionized water (2 mg∙.mL^-1^) were prepared. The appropriate amount of drug injection solution was transferred to a 25mL volumetric flask and filled up to the volume with deionized water to the final concentration of 20 μg∙mL^-1^. These solutions were used for the development and validation of the HPLC method.

### 2.4. Testing the degradation of cytostatics

The procedure for testing the degradation of cytostatics on the surface of reactive sorbents is based on previously published works [23,31,32].

In this procedure, standards were mixed into the decontamination solution in a series of glass vials (Supelco [Bellefonte, PA, USA], 20 mL). At predetermined time intervals (0, 5, 15, 20, 30, 50, 70, 90, and 120 minutes) the reaction was terminated by formic acid (0.1%). Then, the solution was analyzed immediately by HPLC to determine the oxidation or hydrolysis of the transformation products. A similar assay was used to test the degradation of cytostatics on the surface of nanostructured titanium dioxides [33]. In this case, the initial concentration of the cytostatic drug stock solution was 2 mg·mL^-1^, as recommended by the drug manufacturer. Similarly, the reaction was terminated by adding a solution of formic acid (0.1%).

### 2.5. Methods of characterization

For the material characterization of prepared TiO_2_ nanoparticles, physical-chemical methods of analysis were used. The specific surface area and porosity were determined by several point BET method (*Brunauer Emmett-Teller* isotherm); while the pore size distribution (the diameter and pore volume) was determined by *Barret*, *Joyner and Halenda* method (BJH) measured at the liquid nitrogen temperature using an SA-3100 instrument (Beckman Coulter, USA). High resolution scanning electron microscope (HRSEM) Nova NanoSEM 450 (FEI) with Schottky field emission electron source (with a selectable voltage from 1 to 30 kV) and with a BSE (backscattered electrons) and SE (secondary electrons) detection was used for the study of micro- and nanostructures and for the determination of the morphology of prepared materials. An X-ray powder diffraction (XRD) method was used to determine the crystalline composition and crystallite size. Bruker D2 X-ray powder diffractometer with an X-ray lamp (Cu (Kα), 30 kV, 10 mA) and LynxEye one-dimensional detector (1D) (Bruker, Karlsruhe, Germany) was used for this purpose. The diffraction patterns were scanned at 0.00405° and measured in the range of 5° to 95° 2θ. The data was processed in DiffracPlusEva (Bruker AXS) with JCPDS PDF-2 (ICDD, USA) and the structural models from the ICSD database (FIZ Karlsruhe, Germany). Using the Scherrer equation [34] (see Eq 1), the size of the crystallites (a) from the half-width of the diffraction line (FWHM) was determined.

$$a=\frac{K\lambda }{\beta \cos\theta }$$ (1)

The surface degradation reactions (interactions between the sorbent surface and the cytostatic drug) were studied by an *in-situ* DRIFTS technique. For this purpose, Infrared spectrometer Nicolet Impact 400D (Thermo Fisher Scientific, USA) equipped with DRIFTS apparatus The Praying Mantis ^™^ (Harrick) was used. This method allows the study of the mechanism of degradation of cytostatics based on the vibration-rotational states of molecules, or their binding interaction with the sorbent surface [35].

### 2.6. Chromatographic methods

To monitor and identify anthracyclines and their transformation products (TPs), two systems of high-performance liquid chromatography (HPLC) with a diode array detector (DAD) were used: HPLC system Dionex UltiMate 3000 (Thermo Scientific Thermo Scientific ^™^, Palo Alto, USA) and Thermo Finnigan HPLC system (Thermo Scientific ^™^, Palo Alto, USA) with LCQ Fleet ^™^ ion trap electrospray ionizer was used. In the case of chromatographic separation of anthracycline cytostatics and their TPs in the samples obtained from the described experiments, suitable HPLC separation conditions with UV (DAD) detection were found. Subsequently, analyses were performed by HPLC-MS with ESI ionization in an ITMS (ion trap mass spectrometry) mode, for which suitable MS detection conditions with ESI ionization were found. Validation parameters, together with the chromatographic conditions, are detailed in Tables A and B in S1 File.

## 3. Results and discussion

### 3.1. Material characterization of nanocrystalline titanium dioxide

**Fig 2:**
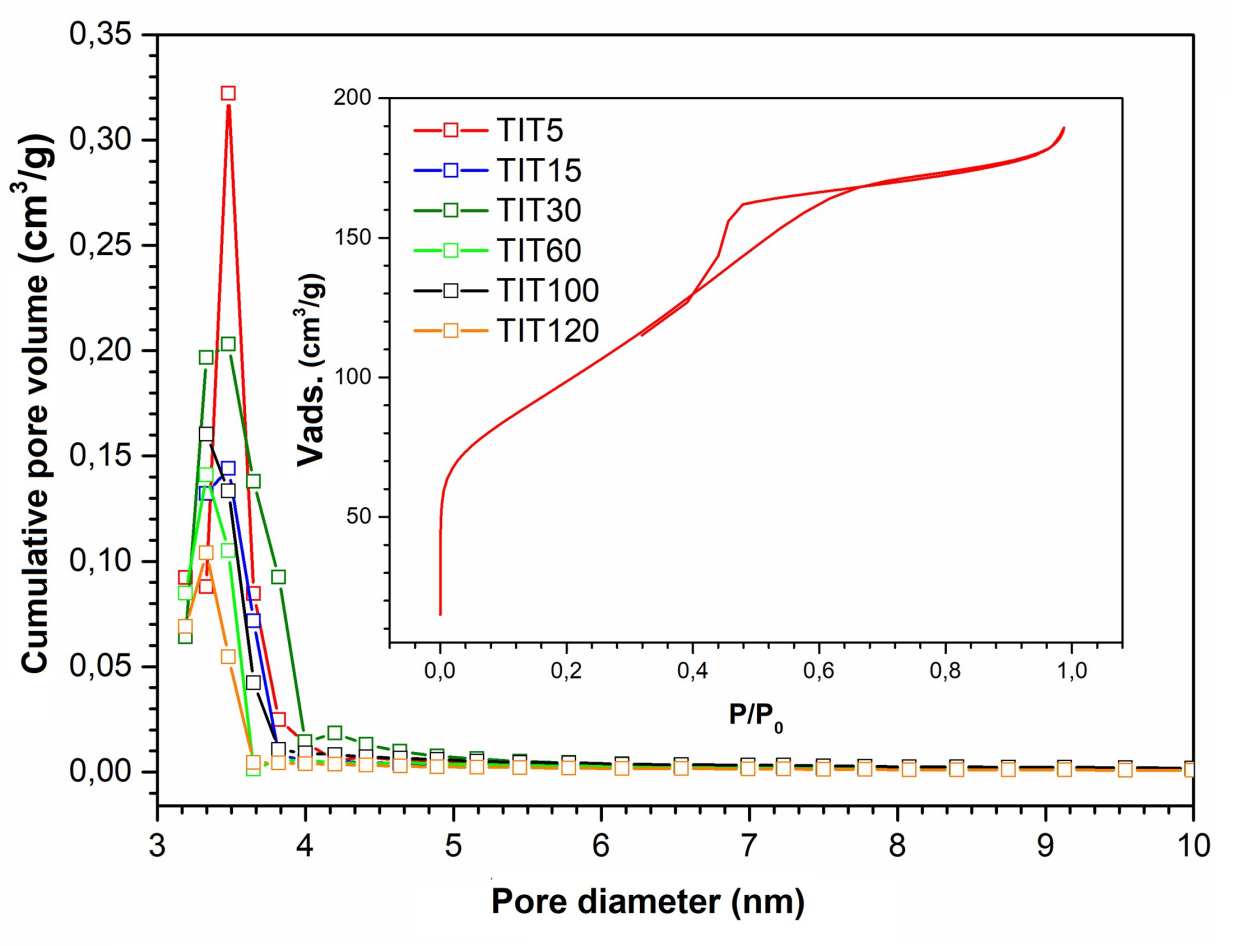
The porosity of TiO_2_ samples. Inset: a hysteresis loop.

Basic procedures for the preparation of reactive sorbents as well as photocatalysts based on nanocrystalline TiO_2_ materials are based on homogeneous precipitation of titanium (IV) oxy-sulfate (TiOSO_4_) with urea (NH_2_)_2_CO [36,37], or thermal hydrolysis of peroxo-complexes [38,39]. The choice of the synthesis method and the appropriate reaction conditions, such as the concentration of the starting materials, the temperature and the pH of the reaction, leads to titanium dioxide materials suitable for reactive adsorption (such as reactive sorbents) but unsuitable for photocatalysis (photocatalysts) [29,40] or vice versa [38]. As can be seen further, homogeneous precipitation of TiOSO_4_ with (NH_2_)_2_CO can be achieved to prepare titanium dioxide with an anatase modification but different texture. A total of six titanium dioxide samples were prepared, different in the amount of titanium oxy-sulfate [5–120 g] (see Table 1). The adsorption-desorption isotherm showed the same hysteresis for all as-prepared samples. The representative isotherm shown in Fig 2 corresponds to Type IV. The shape of the hysteresis loop, which is a transient type of hysteresis of H2 and H3, is related to the filling of empty pores at pressure dependent on the pore size (capillary condensation occurs in mesopores). Pore emptying takes place at lower pressures than their filling. This type is characteristic for nanoparticle samples, with capillary condensation in the interparticle space. All samples have a pore size distribution with a maximum of about 3–4 nm, suggesting, according to IUPAC, the mesoporous character of the materials [41,42]. The specific surface area calculated by the multi-point Brunauer–Emmett–Teller method, total pore volume, as well as the crystallite size of TiO_2_ samples calculated using the Scherrer equation are listed in Table 1. As can be seen in Table 1, the inhibition of the particle size growth with the increasing amount of TiOSO_4_ and the increasing amount of possible amorphous phase manifests itself by an increase in the specific surface area (vary from ~ 305 to 346 m^2^·g^-1^). The total pore volume is almost the same for all samples and ranges from 0.06 (TIT120) to 0.16 cm^3^·g^-1^ (TIT30).

**Fig 3:**
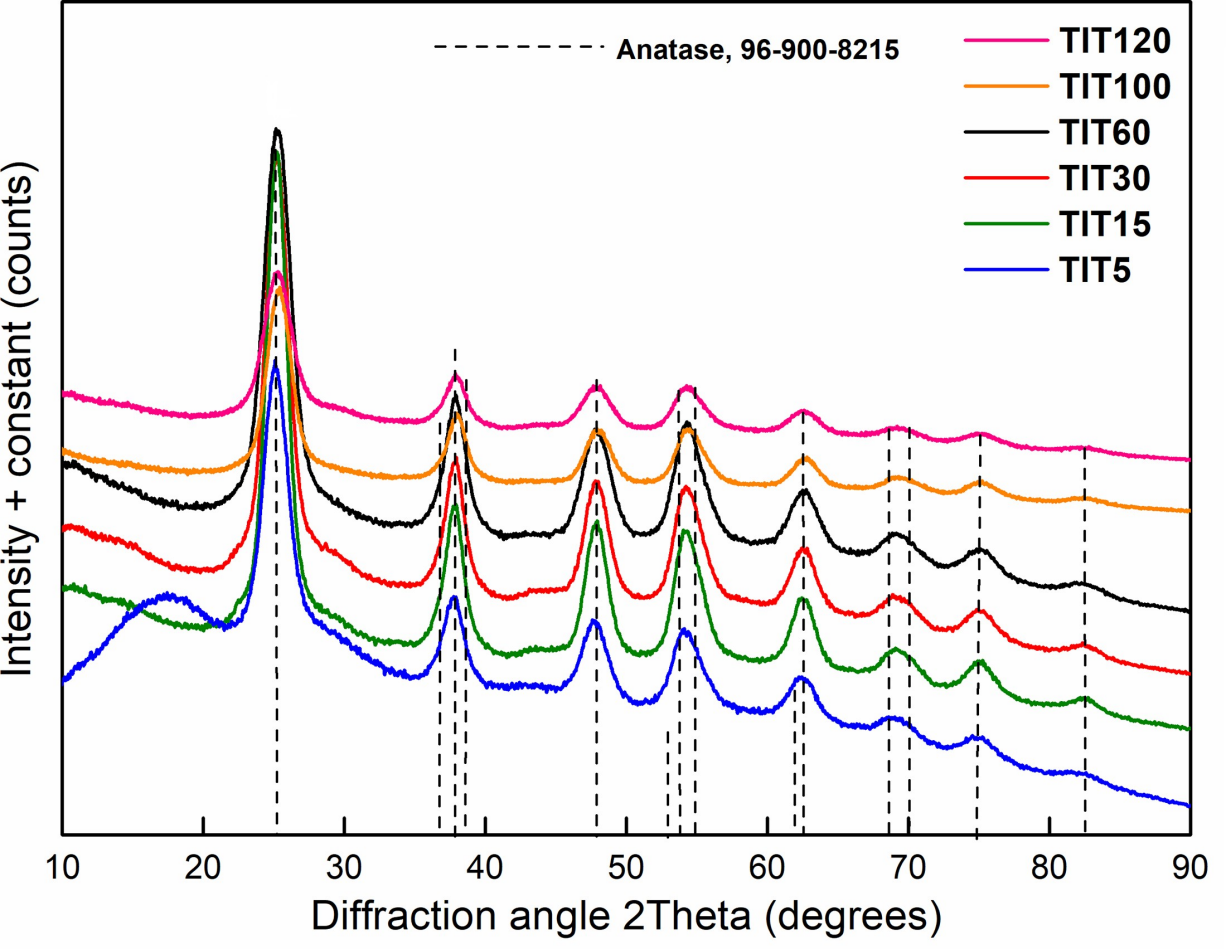
The XRD patterns of the titanium samples.

Diffractograms of TiO_2_ samples (see Fig 3) contain diffraction patterns at a diffraction angle of 2Θ = 25.0, 39.8, 47.8, 54.2, 62.5, 69.0, 75.0 and 82.3°, corresponding to anatase (card 21–1272 in PDF2 ICDD database) [43]. Individual patterns correspond to crystal planes (101), (112), (200), (211), (204), (116), (215) and (303) [44]. The crystallite size was calculated from the most intense peak corresponding to the crystal plane (101) of the diffraction pattern using its half-width. The calculated crystallite size is shown in Table 1 for the individual as-prepared TiO_2_ samples. As can be seen in Table 1, with increasing amounts of added TiOSO_4_, the crystallite size is reduced almost linearly. The decrease in the crystallite size with the increase in TiOSO_4_ concentration in the reaction is probably due to faster nucleation, i.e., the formation of more crystallization nuclei.

It is well known that homogeneous precipitation of metal salts with urea leads to the formation of crystalline nanoparticles of metal oxides with spherical morphology [45–47].

**Fig 4:**
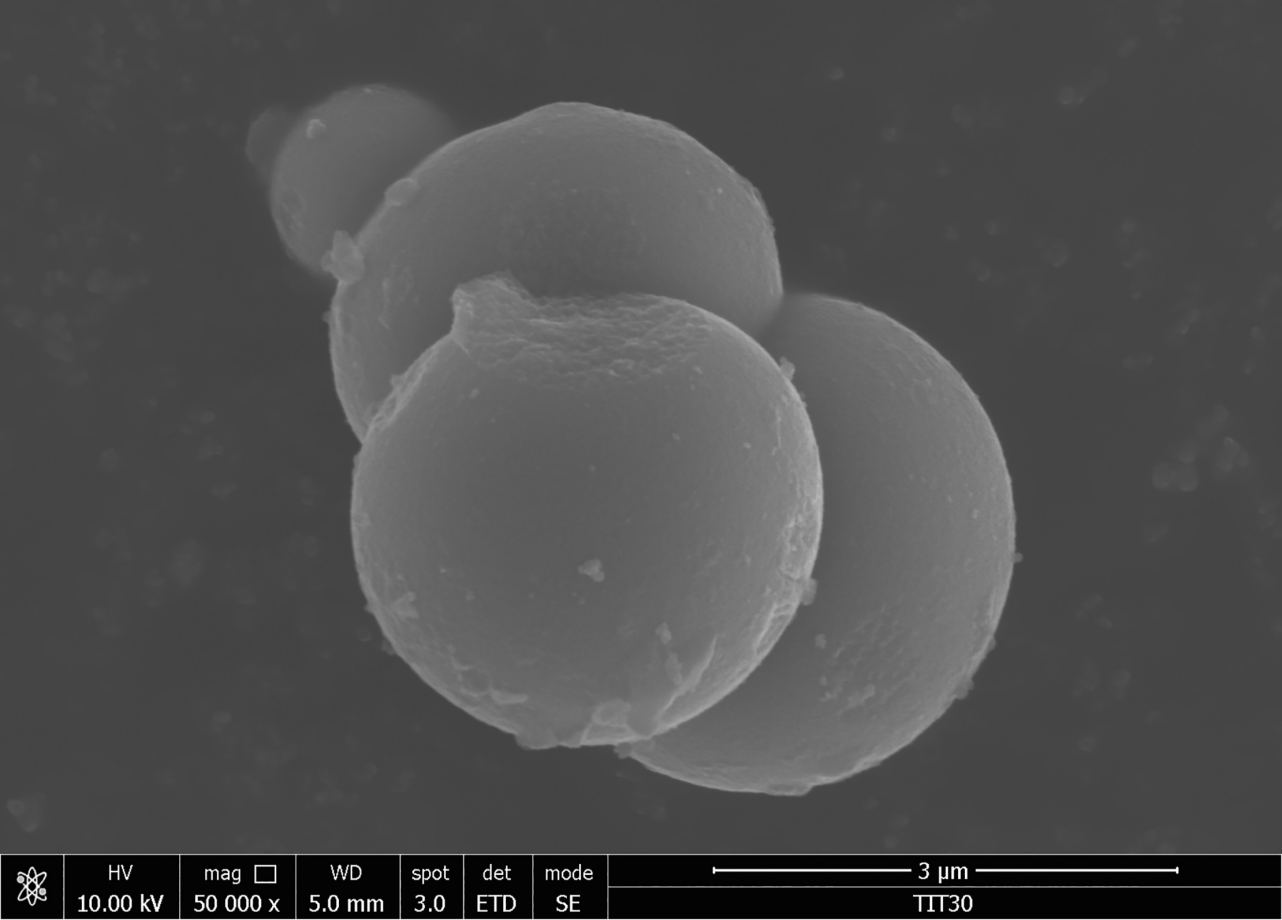
HRSEM image of sample TIT30 prepared by homogeneous precipitation with an initial amount of 30 g TiOSO_4_.

**Fig 5:**
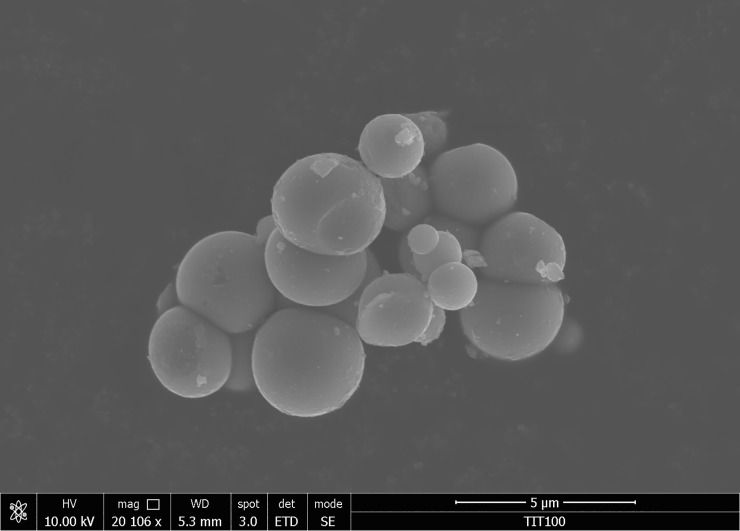
HRSEM image of sample TIT100 prepared by homogeneous precipitation with an initial amount of 100 g TiOSO_4_.

The formation of spherical agglomerates is related to the effect of minimizing surface energy. From the viewpoint of minimizing the surface energy of nanoparticles, the sphere is geometrically optimal (an effort to assume the smallest possible surface). The final shape of the particles then depends on whether the so-called oriented or isotropic aggregation is applied during the synthesis. Among the various factors that control the aggregation, dispersion forces, and electrostatic interactions between the primary nanoparticles are the most important. In the case of isotropic aggregation (which usually leads to the formation of spherical particles) this effect occurs near the so-called isoelectric point (at a pH of the zero Zeta potential; in the case of TiO_2_ it is pH ~ 6.5–7.5) [48]. Samples prepared by the homogeneous precipitation method are formed by spherical agglomerates of 3–5 μm agglomerates with a narrow particle size distribution and good homogeneity, as shown in Fig 4. As can be seen in the HRSEM micrograph, the individual nanoparticles form agglomerates and clusters resemble a grape. Samples prepared with lower TiOSO_4_ loading values (5–60 g) show the indicated morphology. The opposite is the second type of material, composed of separate particles with excellent crystallinity and mesoporous texture at the microporous boundary (~ 3 nm), forming samples prepared with an added amount of TiOSO_4_ ranging from 100 to 120 g (see Fig 5).

### 3.2. Evaluation of cytostatic degradation in decontamination agents

**Fig 6:**
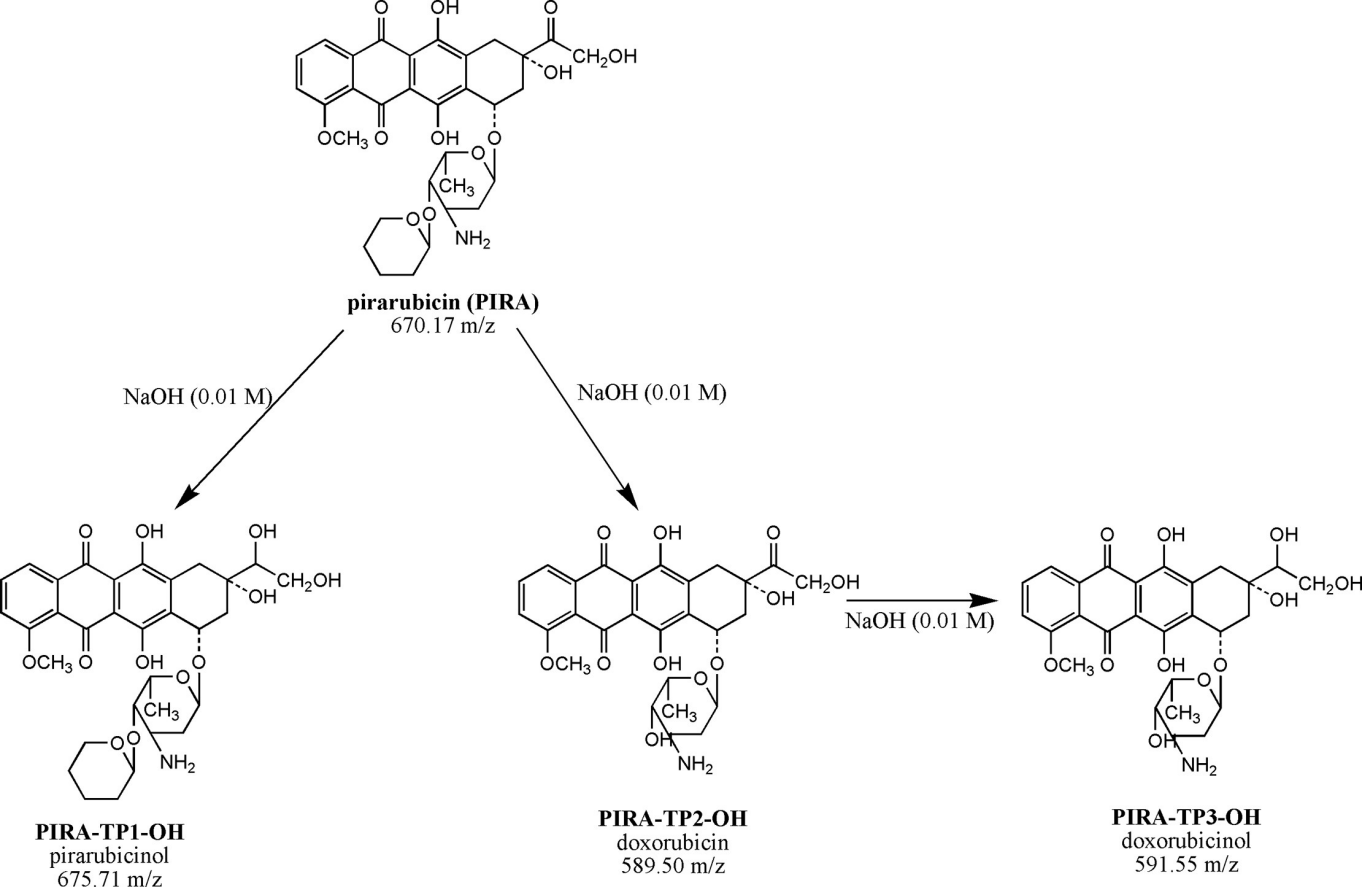
Scheme of PIRA degradation after its reaction with a solution of 0.01 M NaOH. Abbreviations: PIRA, pirarubicin; PIRA-TP1-OH, pirarubicinol, PIRA-TP2-OH doxorubicin, PIRA-TP3-OH, doxorubicinol.

**Fig 7:**
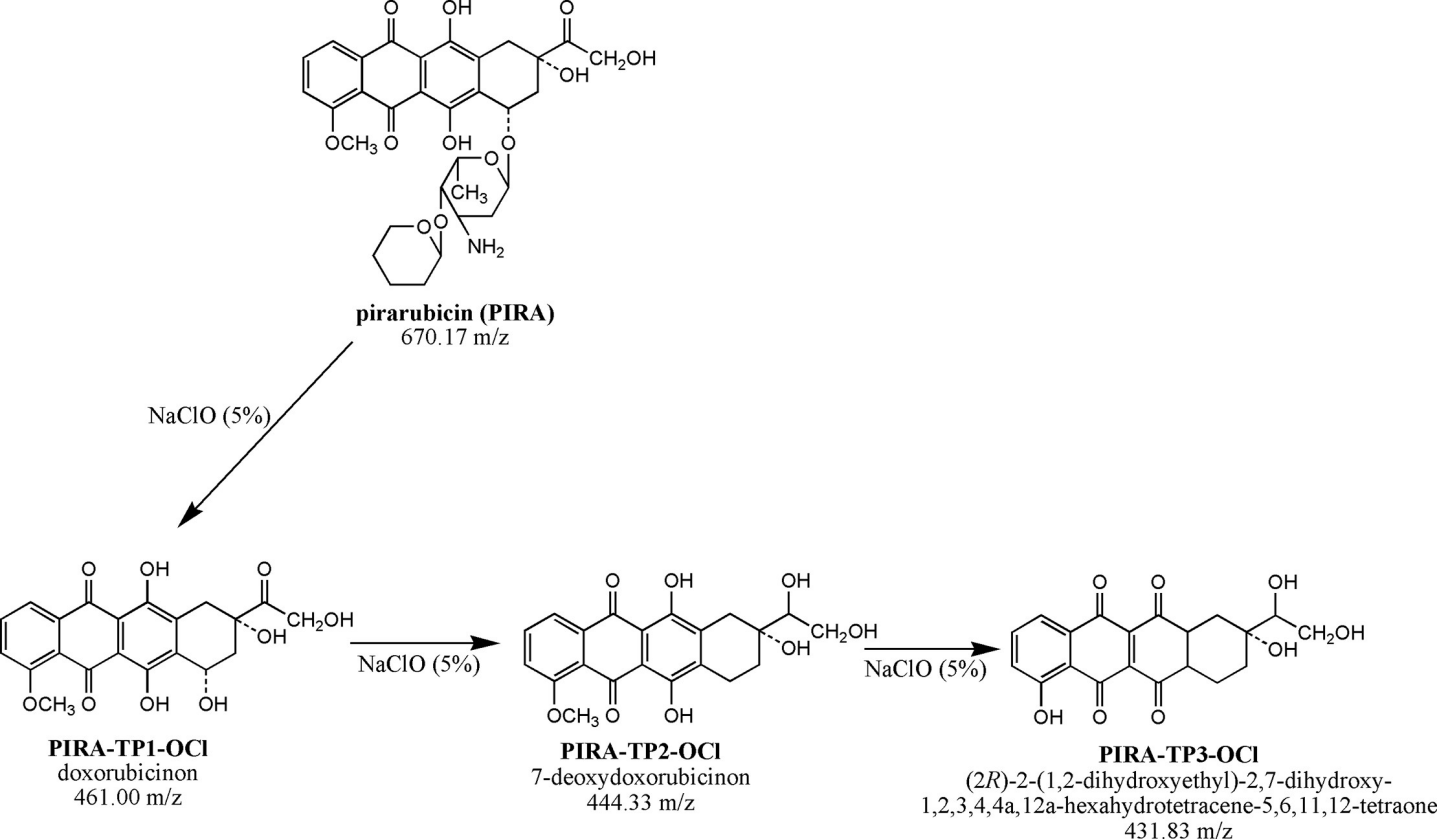
Scheme of PIRA degradation after its reaction with a solution of 5% NaClO. Abbreviations: PIRA, pirarubicin; *PIRA-TP1-OCl*, doxorubicinon, *PIRA-TP2-OCl*, 7-deoxydoxorubicinon, *PIRA-TP3-OCl*, (*2R*)-2-(1,2-dihydroxyethyl)-2,7-dihydroxy-1,2,3,4,4a,12a-hexahydrotetracene-5,6,11,12-tetraone.

In the case of the degradation of pirarubicin (670.17 m/z) in an alkaline agent, NaOH (0.01 M) three transformation products (TPs) were identified: Pirarubicinol (*PIRA-TP1-OH*), doxorubicin (*PIRA-TP2-OH*) and doxorubicinol (*PIRA-TP3-OH*). All three of these TPs are among the most important metabolic products commonly identified in the blood plasma of patients who ingested pirarubicin [49]. The scheme of pirarubicin’s decomposition in the NaOH decontamination agent is presented in Fig 6. Another case occurred in a sodium hypochlorite solution (5%). In this case, three TPs have been identified to have been formed by the redox and hydrolysis reaction. The first TP denoted as *PIRA-TP1-OCl* was identified as adriamycinone (doxorubicinon; 461.00 m/z) generated by the hydrolytic deglycosidation of pirarubicin. Furthermore, adriamycinone was transformed by a reductive deglycosidation into an intermediate identified as 7-deoxyadriamycinone (*PIRA-TP2-OCl*, 444.33 m/z) except for the TP designated as *PIRA-TP3-OCl* (431.83 m/z), which is formed by the demethoxylation of adriamycinone [50]. The scheme of the decomposition of pirarubicin in an NaClO solution is presented in Fig 7.

**Fig 8:**
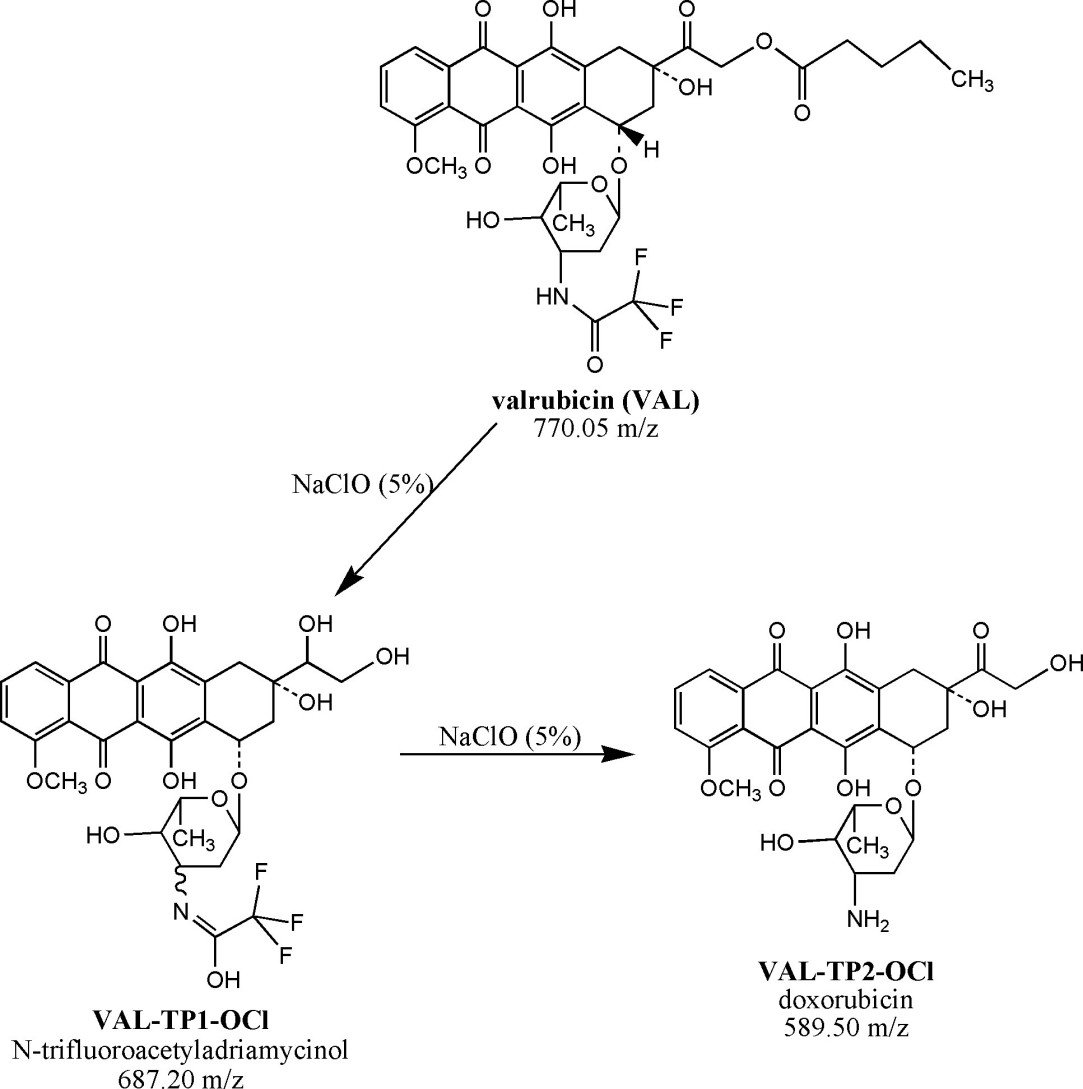
Scheme of VAL degradation after its reaction with a solution of 5% NaClO. Abbreviations: VAL, valrubicin; *VAL-TP1-OCl*, N-trifluoroadriamycinol, *VAL-TP2-OCl*, doxorubicin.

**Fig 9:**
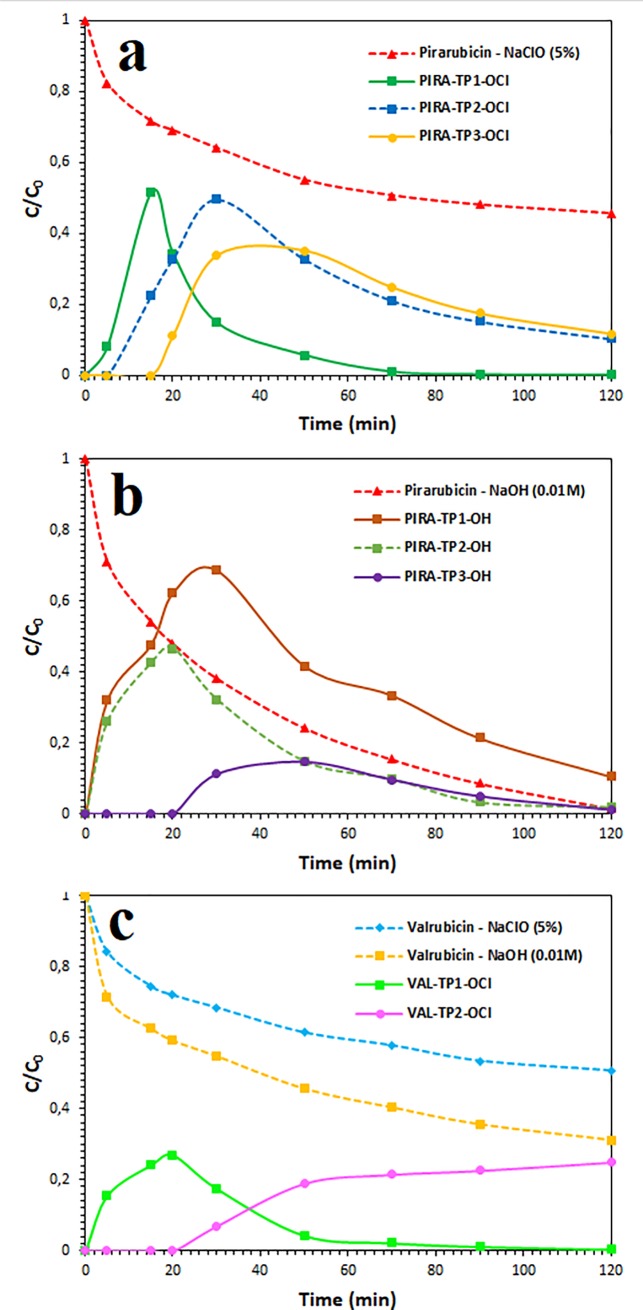
Transformation products formed during the degradation of anthracyclines plotted as a function of the normalized concentration (*C*/*C*_0_) of pirarubicin and valrubicin decay. Note: Transformation products formed during the decontamination of pirarubicin in the presence of (a) 5% NaClO agent, (b) 0.01 M NaOH agent; and the decontamination of valrubicin in the presence of 5% NaClO agent (c).

In the case of the degradation of valrubicin (770.05 m/z) in a sodium hypochlorite solution (5%), three TPs were identified: The first TP denoted as *VAL-TP1-OCl* (687.20 m/z) was identified as N-trifluoroacetyladriamycinol formed by the oxidation of the ketone portion of the trifluoroacetyl group and by a cleavage valerate from the anthraquinone cycle. The second TP, doxorubicin (*VAL-TP2-OCl*; 589.50 m/z), was formed by the cleavage of the trifluoroacetyl group of the valrubicin molecule. The proposed scheme of valrubicin degradation is shown in Fig 8. No transformation product was identified after the reaction of valrubicin with sodium hydroxide (0.01 M). The formation of all TPs is collected in Fig 9A, 9B and 9C.

As can be seen in our previous results [23], in the case of chemical degradation of doxorubicin (DOX) in the presence of NaClO (5%) solution, we detected three transformation products: 4-amino-6-methyltetrahydro-2H-pyran-2,5-diol (hydroxyl-aminosugar) after 5 min, 4-amino-6-methyltetrahydro-2H-pyran-2,3,5-triol (hydroxyl-aminosugar) after 20 min, and 5,12-dihydroxy-7- methoxy-2,3-dihydrotetracene-1,4,6,11-tetraone after 50 min (as the most stable final transformation product). Similarly, in the 0.01 M sodium hydroxide solution, during 90 minutes we detected four reaction products from doxorubicin: 10-methoxy-6,11-dihydrotetracene-1,3,5,6,11,12-hexaol after 15 min, 6-methoxy-5,10-dihydro-1H-cyclopenta[b]anthracene1,2,4,5,10,11-hexaol after 20 min, 10-hydroxy-6-methoxy-4-methyl1H-cyclopenta[b]anthracen-5(10H)-one after 50 min, and 4Hcyclopenta[b]anthracen-9(8H)-one after 90 min (as the most stable final transformation product).

The extracted chromatograms of individual TPs of pirarubicin and valrubicin in both decontamination agents are shown in Figures A-H in S1 File. It is clear from the mass spectra of ions formed by the ionization of analytes of anthracycline cytostatics that the molecules are ionized in ESI to adductic ions (adducts with matrix) [M+HCO_2_H]^+^. The list of major ions of cytostatics, as well as the retention times (t_R_, min) and proposed elemental composition, are reported in Table 2.

**Table 2:** Summary of LC-MS data, together with the molecular formula of cytostatics and transformation products (TPs).

[M+HCO_2_H]^+^ (molecular formula)	Compound name	t_R_ (min)	Agent (concentration)
*m/z* 670,17 (C33H39NO11)	Pirarubicin (PIRA)	1.71	NaOCl (5%)
*m/z* 461,00 (C21H18O9)	PIRA-TP1-OCl	3.38	
*m/z* 444,33 (C21H20O8)	PIRA-TP2-OCl	2.47	
*m/z* 431,83 (C20H18O8)	PIRA-TP3-OCl	3.59	
*m/z* 675,71 (C_32_H_39_NO_12_)	PIRA-TP1-OH	2.89	NaOH (0.01M)
*m/z* 589,50 (C_27_H_29_NO_11_)	PIRA-TP2-OH	3.76	
*m/z* 591,55 (C_27_H_31_NO_11_)	PIRA-TP3-OH	4.22	
*m/z* 770,05 (C_34_H_36_F_3_NO_13_)	valrubicin (VAL)	3.16	NaOCl (5%)
*m/z* 687,20 (C_29_H_30_F_3_NO_12_)	VAL-TP1-OCl	3.55	
*m/z* 589,50 (C_27_H_29_NO_11_)	VAL-TP2-OCl	3.75	

### 3.3. Evaluation of anthracycline removal on a titanium dioxide surface

In this section, the adsorption activity of TiO_2_-based sorbents with different amounts of TiOSO_4_ added to the synthesis (TIT5-TIT120) was tested for the reaction with anthracycline antibiotics (represented by pirarubicin and valrubicin). In the case of sorbent activity, the initial cytostatic concentration was measured by an HPLC-DAD method. The formation of transformation products (TPs) during the adsorption was verified by LC-MS. The results of the adsorption kinetics of selected cytostatic drugs on TiO_2_ sorbents were processed by *OriginPro 9*.*0*.*0* software (OriginLab Corp., Northampton, MA 01060 USA). The kinetic curves (the time dependence of adsorption on the titania surface) were fitted using a kinetic equation Eq (2) for the first-order kinetics reaction [47]:

$$-\frac{dc}{dt}=k_{1}\left(c_{0}-c\right)+k_{2}\left(c_{0}-c\right)+A$$ (2)

where *c* is the current concentration of cytostatics over time, *c*_*0*_ is the initial concentration of cytostatics and *k*_*1*_, *k*_*2*_ are the rate constants (min^-1^). *A* is a constant expressing the unreacted proportion of cytostatics at the end of the reaction.

**Fig 10:**
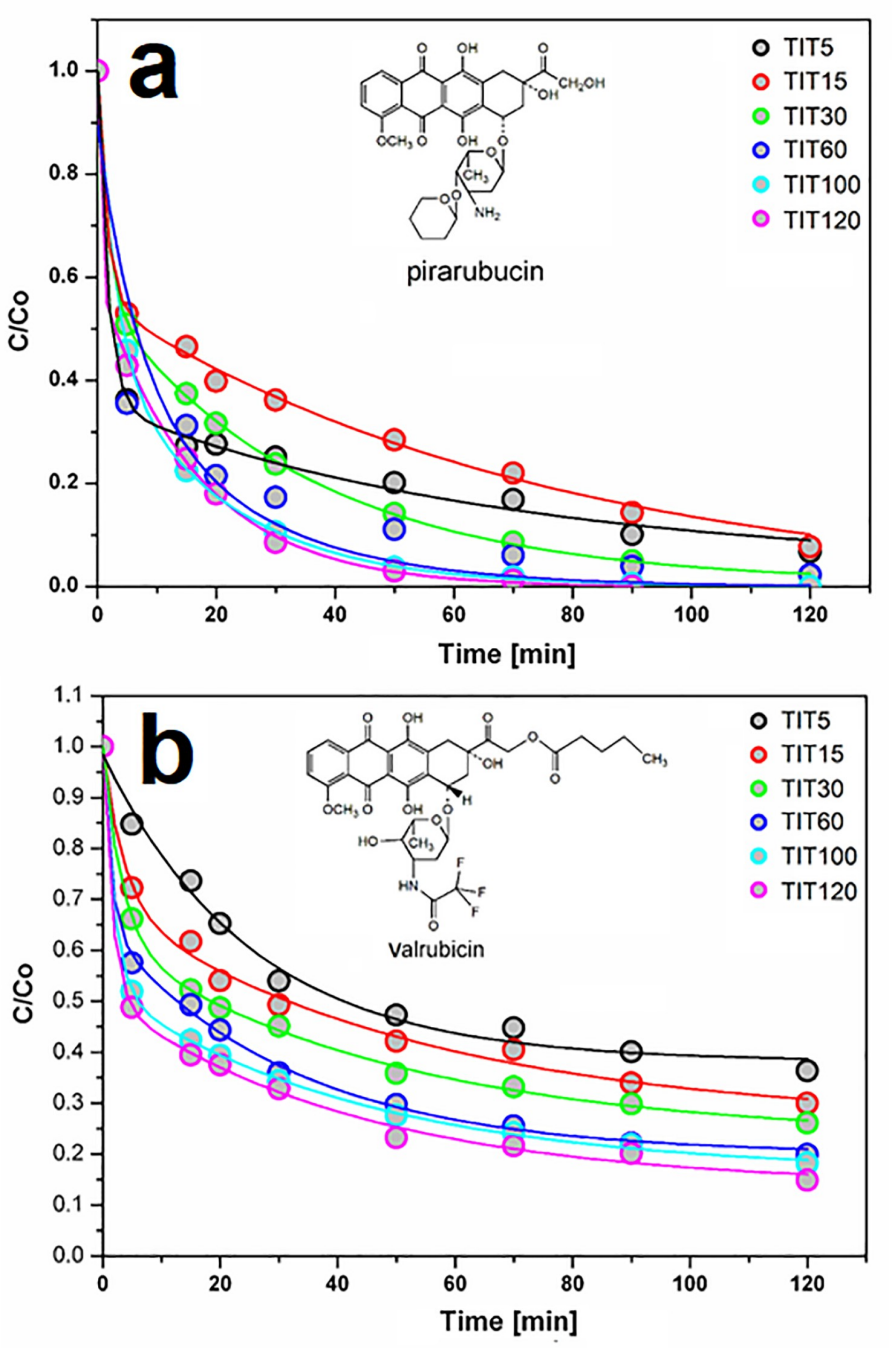
Normalized kinetic curves of pirarubicin (a) and valrubicin (b) adsorption on TiO_2_ samples differing in TiOSO_4_.

The normalized kinetic curves of pirarubicin and valrubicin adsorption are shown in Fig 10A and 10B.

In all cases, kinetic parameters such as the reaction rate constant (min^-1^) and the half time of conversion (min) were determined. Using the DRIFTS technique, the mechanism of drug interaction with a titanium oxide surface was verified. Based on LC-MS and DRIFTS measurements, the possible mechanisms of cytostatic drug degradation on TiO_2_ samples were proposed.

The kinetic parameters expressed by the rate constants k_1_ and k_2_ (min^-1^) of the anthracycline cytostatics adsorption on TiO_2_ sorbents are shown in Table 3.

**Table 3:** Parameters of the pseudo-first-order kinetic model for the adsorption of anthracycline cytostatics on TiO_2_ sorbents.

Sample	Valrubicin		Pirarubicin	
	k_1_ [min^-1^]	k_2_ [min^-1^]	k_1_ [min^-1^]	k_2_ [min^-1^]
TIT5	0.040^a^	0.040^a^	0.055	0.009
TIT15	0.290	0.019	0.651	0.013
TIT30	0.315	0.021	0.605	0.029
TIT60	0.745	0.032	0.156	0.045
TIT100	0.537	0.024	0.356	0.045
TIT120	0.633	0.232	0.890	0.060

^a^Adsorption process and chemical interaction are in equilibrium.

As can be seen in the results shown in Table 4, the total adsorption of pirarubicin (~ 2 mg ml^-1^) on a TiO_2_ surface prepared from 120 g of titanyl sulfate (sample TIT120) occurred over 5 minutes with the conversion rate of ~ 100%, as is illustrated by the value of the rate constant k_1_, which ranged from ~ 0.9 min^-1^ (corresponding to a half-life of ~ 1 min). In the case of valrubicin adsorption, the highest activity (in terms of a reaction rate) was achieved by a sample prepared with an input amount of 60 g of titanyl oxy-sulfate (sample TIT60). The highest degree of conversion (~ 85%) for valrubicin was then measured on a sample prepared from 120 g of titanyl sulfate (TIT120 sample). The results of the kinetics of the adsorption of both anthracyclines on the surface of TiO_2_ samples show a different binding of anthracyclines to the substrate that may be due to the presence of different functional groups in the anthracycline molecules. For example, an impaired TiO_2_ activity in the case of valrubicin interaction may be due to the presence of a stable trifluoroacetyl group attached to the daunosamine sugar unit, where it blocks the amino group during its interaction of the amino group with the hydrophilic surface of titanium oxide [51].

**Table 4:** Assignment of vibration modes of interaction of anthracycline cytostatics with a titanium surface.

Measured wave number [cm^-1^]	Vibration mode	Values of wave numbers taken from the literature [cm^-1^]	Assignment
971	*ν*(C-O-C)	1180–950	Symmetrical valence vibrations of the etheric group in ethers
1434	Amide II	1525–1435	C–N stretching coupled with N-H bending modes
1635	*ν*(O-H)	1640–1635	Symmetrical valence vibrations in free O-H groups in a water molecule
3224	*ν*(O-H)	3300–2500	Symmetrical valence vibrations in O-H groups

**Fig 11:**
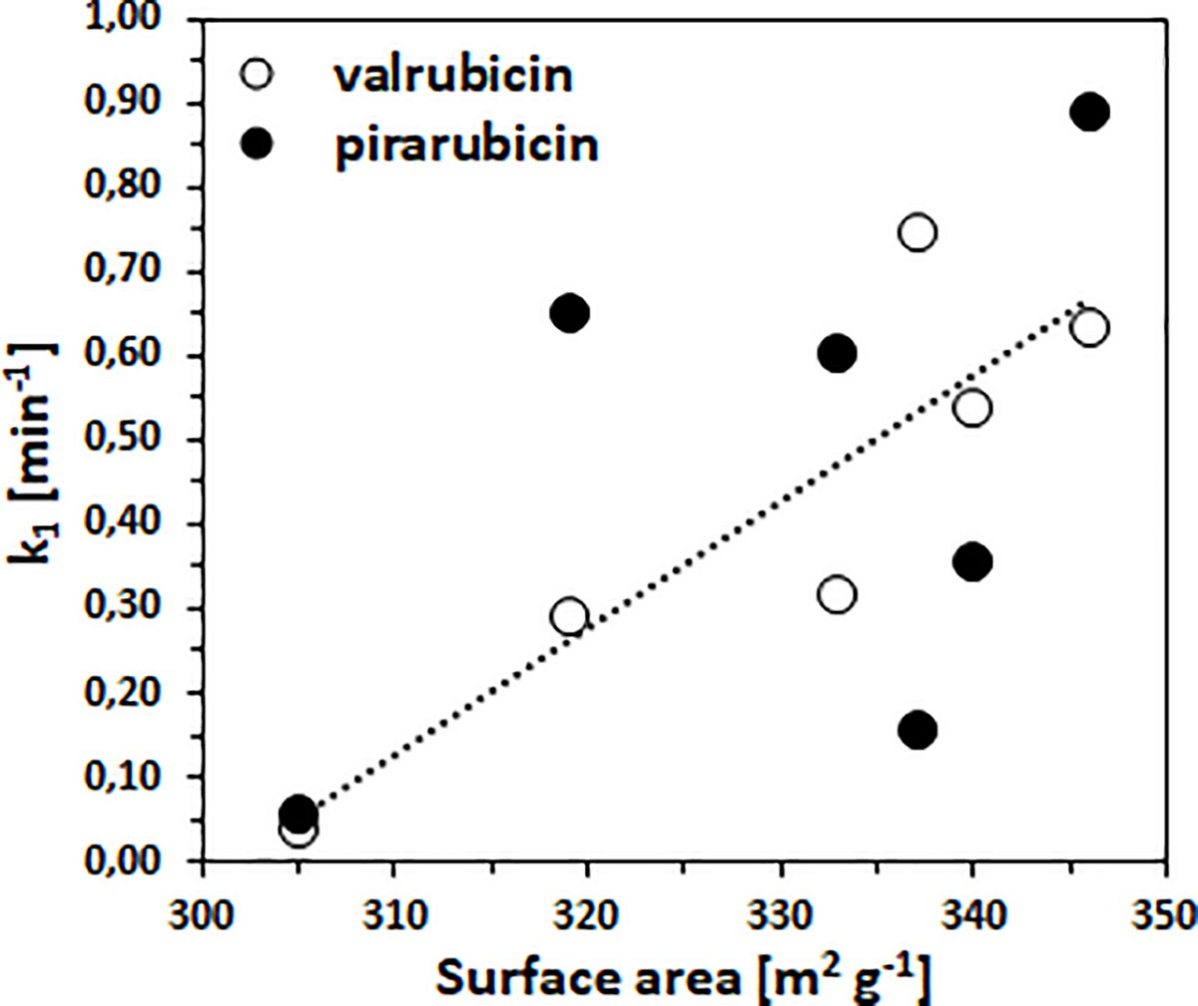
Dependence of k_1_ (min^-1^) versus surface area (m^2^ g^-1^) of prepared TiO_2_ samples.

As can be seen in our previous results [23], in the case of doxorubicin adsorption, the degradation product daunosamine (with a product ion of 148.09 m/z) was identified in the solution by LC-MS by the cleavage of the glycoside bond in the doxorubicin molecule. The resulting doxorubicinone unit was then firmly attached to the TiO_2_ surface *via* hydrogen bridges formed by their interaction with surface titanium dioxide hydroxyls. Fig 11 shows the dependence of the rate constant k_1_ on the surface area of the prepared TiO_2_ samples. As can been seen, in the case of valrubicin adsorption, with the increasing surface area, the rate constant k_1_ grows until the surface area around 337 m^2^ g^−1^ (correspond to 60 g of TiOSO_4_) is reached, suggesting the ideal surface area for the performance of valrubicin removal. A quite different situation was observed in the case of pirarubicin removal, where no correlation between the rate constant and the surface area was found. The highest activity was found for a sample with 120 grams of TiOSO_4_ (TIT120).

**Fig 12:**
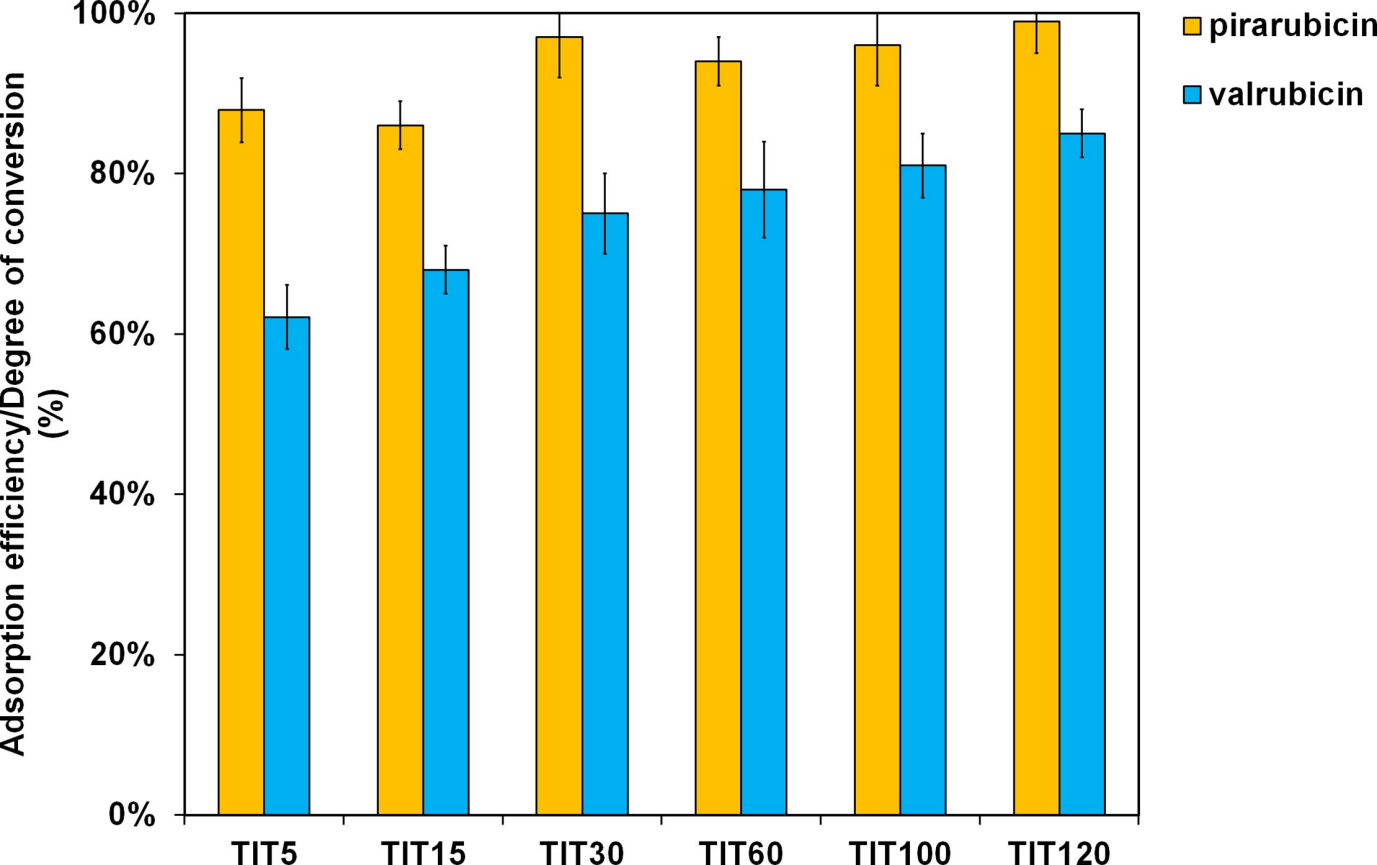
The degree of conversion of pirarubicin (a) and valrubicin (b) using the nanostructured TiO_2_-based adsorbents after 120 minutes.

The adsorption efficiency/degrees of conversion of pirarubicin and valrubicin after 120 minutes are compared in Fig 12.

#### 3.3.1. Adsorption/interaction mechanism of anthracyclines on TiO_2_ nanoparticles

It is generally known that some nanocrystalline forms of oxides of alkaline earth metals (Mg and Ca) [52] and light metals (Al and Ti) [53] have the ability to react with chemical warfare agents (CWA’s) such as sulfur mustard (HD), soman (GD) and VX agent [36,54]. It turns out that nanocrystalline metal oxides may have a high potential for detoxifying effects on cytostatics. This hypothesis was made on the basis of knowledge about the decomposition of mustard poisoning substances using nanoparticles of titanium oxides [32]. Cytostatics of the oxazophosphine family are derived from nitrogen mustards and are subject to the same adsorption and degradation phenomena [55]. These mechanisms appear to be useful for other classes of cytostatics, such as anthracycline antibiotics. The binding sites on the anthracyclines are found on the chelating quinone and the phenolic acids located on both sides of the anthracycline aromatic groups [56]. Currently, titania nanostructures are mainly used as drug delivery systems (DDS). In most cases, nanocrystalline titanium dioxide can be synthesized by wet or dry processes. In last years, a variety of methods, such as sol-gel [57,58], hydrothermal/solvothermal methods [59,60], or methods based on electrochemical anodization [61–63] have been developed to control the size, morphology, and porosity and of the resulting TiO_2_ nanostructures. DDS methods have been developed primarily for the anthracycline cytostatic doxorubicin, which is one of the most commonly used cytostatics. Ren *et al*. (2013) modified TiO_2_ nanoparticles with doxorubicin (DOX). As it turned out, the electrostatic interactions hold the DOX and nanoparticles together [64]. In another work, Qin *et al*. (2011) synthesized highly water-dispersible TiO_2_ nanoparticles with abundant carboxyl groups using a ligand exchange method. Briefly, the oleic acid coating on the surface of TiO_2_ was exchanged with carboxylic silane and modified nanoparticles were used for the surface interaction of DOX [65]. Recently, most of the researchers described the adsorption (or in conjunction with photocatalytic degradation) of ibuprofen on the titania surface from the solution [66,67].

Another use of titania nanoparticles in DDS was proposed by Galkina *et al*. (2015). They engaged in preparation of a cellulose nanofiber–titania nanocomposite for the DDS of two drugs: Tetracycline (TC) and phosphomycin (PHOS). The results showed that different modifications of the titania nanocomposite in interaction of the drugs lead to differences in the kinetics of drug release. It was found that PHOS molecule binds to titania *via* a complexation process which results in the formation of the complex that is very stable. On the other hand, TC is bonded to the surface of titania by forming quite stable phenoxide complexes [68]. The mechanism of dexamethasone interaction into nanotubular TiO_2_ was proposed by Zhang *et al*. (2014). The amount of drug-loaded in nanopores was determined using UV/Vis spectrophotometer and Fourier-transform infrared spectroscopy (FTIR). FTIR measurements showed that the hydrogen bonds are formed between drug molecules and the surface of TiO_2_ film [69].

**Fig 13:**
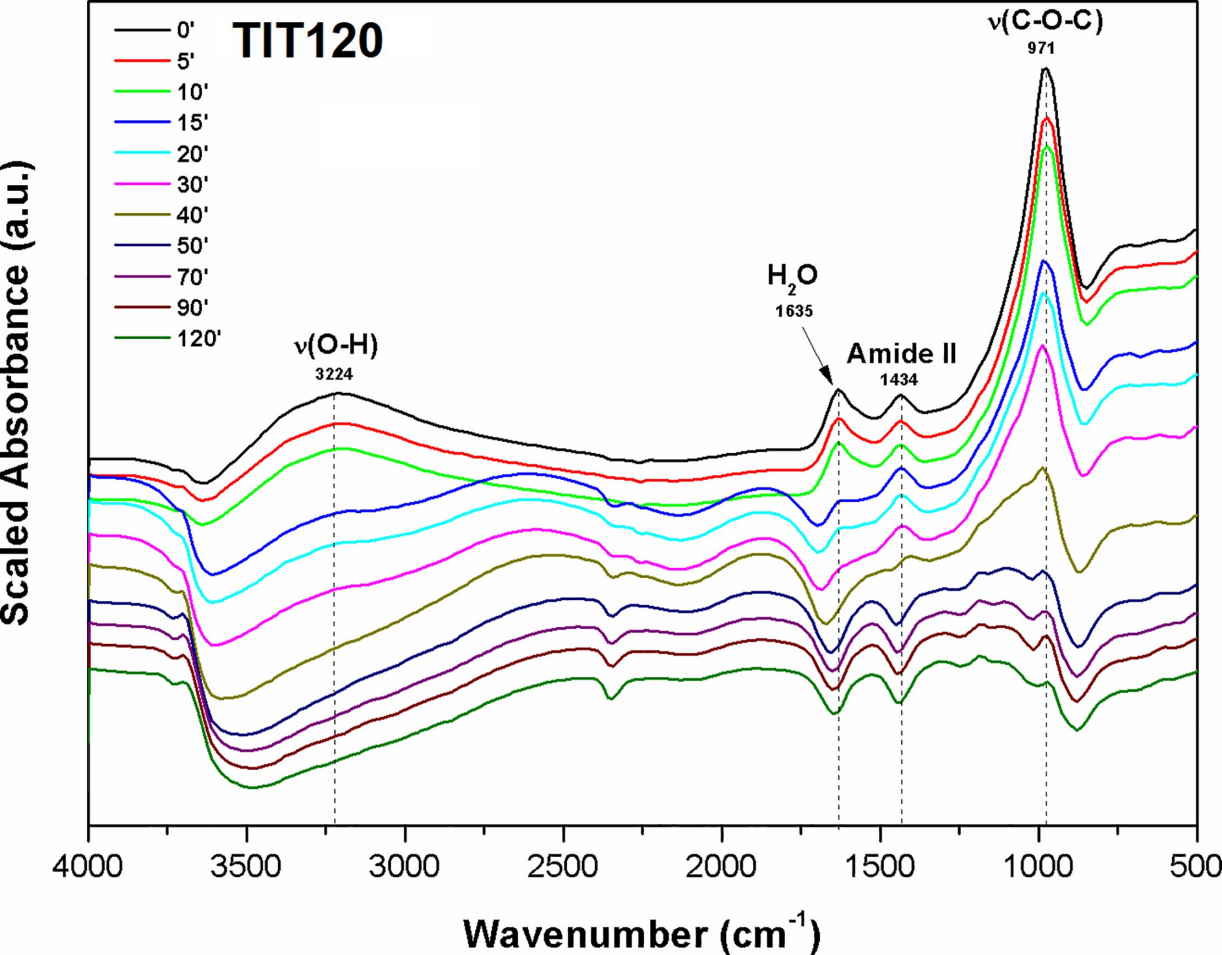
DRIFTS spectra of pirarubicin interaction with the TiO_2_ surface (TIT120).

**Fig 14:**
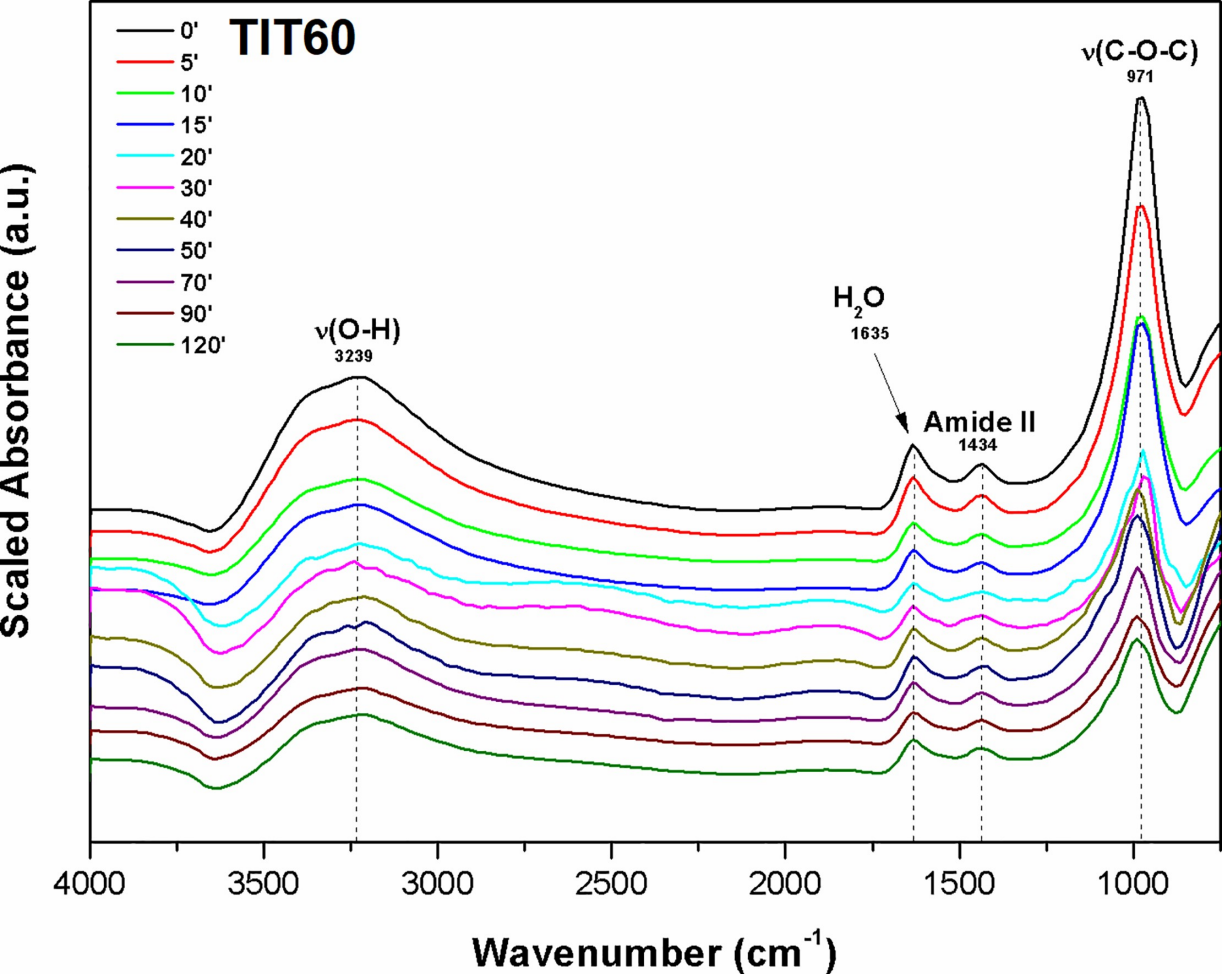
DRIFTS spectra of valrubicin interaction with the TiO_2_ surface (TIT60).

In our case, the adsorption interaction of both anthracyclines on TiO_2_ nanoparticles has been studied by means of DRIFTS technique. Figs 13 and 14 show DRIFTS spectra obtained by kinetic scanning of the interaction of pirarubicin (or valrubicin, respectively) with the sample surface of TIT120 (or TIT60 respectively).

The individual band assignments were based on available literature [70–72] and are presented in Table 4.

As can be seen in Figs 13 and 14, DRIFTS spectra show the disappearance of the broadband C-O-C group (~ 970 cm^-1^) during the anthracyclines interaction with the TiO_2_ surface. The disappearance of these bands is associated with the cleavage of the glycoside bond in the anthracycline molecule during the cleavage of the daunosamine unit. The interaction of the resulting doxorubicinone unit with the TiO_2_ surface *via* hydrogen bridges is confirmed by the disappearance of the broad O-H band in the wavelength region of ~ 3200 cm^-1^ (OH groups involved in the drug interaction). Weak bands with wavelengths of about 1430 cm^-1^ monitored upon the drug interaction correspond to C–N stretching coupled with N-H bending modes (Amide II band). The disappearance of these bands points to the interaction of the NH group with the oxygen atoms (O^2-^) in the metallic lattice [73]. Free unsaturated ions O^2-^ in the crystal lattice of nano-oxides act as strong Bröndsted bases [74]. From these DRIFTS results, it can be evident that binding of anthracyclines to the surface of titanium oxide occurs via the electrostatic interaction of protonated–NH_3_^+^ bonds and unsaturated ions O^2-^ in the crystal lattice and hydrogen bonding between–OH groups of anthracyclines and surface–OH groups of titanium dioxide.

**Fig 15:**
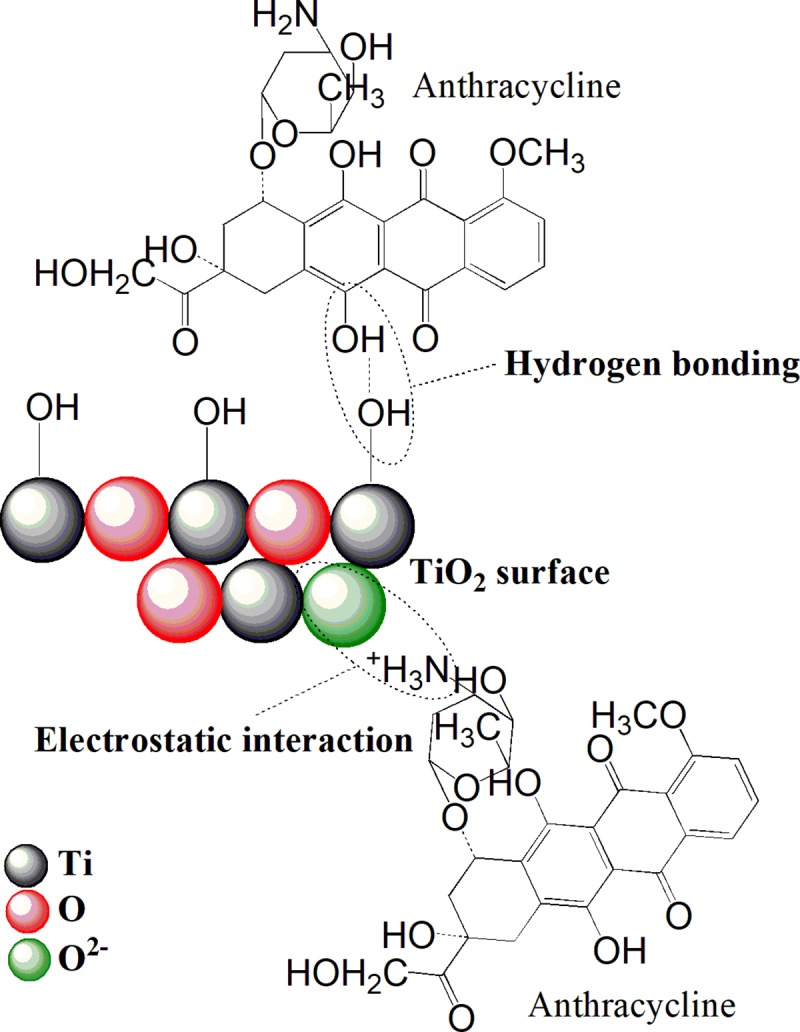
The scheme of the adsorption interaction between anthracycline molecules and TiO_2_ nanoparticles.

In the case of valrubicin, the presence of a trifluoroacetyl group based on DRIFTS measurements was not confirmed. A similar reduction in the broadband intensity of the C-O-C group (~ 970 cm^-1^) during the adsorption on the TiO_2_ surface was confirmed in this case as well. The presence of this band, even after the reaction time of 120 min, is probably due to the participation of the trifluoroacetyl group in the valrubicin adsorption. All proposed interactions of an anthracycline with a titanium surface are presented in Fig 15.

## 4. Conclusions

In this study, two anthracycline cytostatics (PIRA and VAL) were subjected to the process of forced degradation in the presence of decontamination agents (sodium hydroxide and sodium hypochlorite) as the representative alkali and oxidizing agents. The tested cytostatics were degraded to several transformation products (TPs) in both agents at room temperature, which were separated and identified by LC-MS *in-situ*. It was found that VAL is stable in the solution of sodium hydroxide (0.01M). In contrast, an adsorbent based on nanocrystalline titanium oxide prepared *via* homogeneous precipitation with urea can bind the drug on its surface. As can be seen in the results, titanium oxide often adsorbs anthracyclines during the first 5 minutes with an efficiency of about 100%. The results of anthracycline adsorption kinetics on the surface of TiO_2_ samples revealed a different anthracycline binding to the substrate, which is due to the presence of different functional groups in the anthracycline molecules. Based on the *in-situ* DRIFTS measurements, it can be concluded that nanostructured titanium dioxide is suitable for safely capturing and removal of anthracycline cytostatics under mild conditions.

## Supporting information

S1 File Further details for chromatographic measurements (Table A), HPLC method validation (Table B) and supplementary figures: LC-MS chromatograms (Figures A-C, E, and F) and mass spectra (Figures D, F, and G).
(DOCX)
