# Supplementary material for: Removal of anthracycline cytostatics from aquatic environment: Comparison of nanocrystalline titanium dioxide and decontamination agents
Source: PLoS One. 2019 Oct 11;14(10):e0223117. doi: 10.1371/journal.pone.0223117 (PMC6788709; doi:10.1371/journal.pone.0223117)
Supplement: S1 File — Further details for chromatographic measurements (Table A), HPLC method validation (Table B) and supplementary figures: LC-MS chromatograms (Figures A-C, E, and F) and mass spectra (Figures D, F, and G). (DOCX) [file pone.0223117.s001.docx]

**Supporting Information**

Removal of anthracycline cytostatics from aquatic environment: Comparison of nanocrystalline titanium dioxide and decontamination agents

Martin Šťastný^1*^, Václav Štengl^1^, Irena Štenglová-Netíková^3^, Michaela Šrámová-Slušná^1^ and Pavel Janoš^2^

^1^Institute of Inorganic Chemistry of the Czech Academy of Sciences, 25068, Řež, Czech Republic

^2^Faculty of the Environment, J.E.Purkyně University in Ústí nad Labem,

Ústí nad Labem 400 96, Czech Republic

^3^1^st^ Faculty of Medicine, Charles University in Prague, Ovocný trh 3-5,

116 36 Praha 1, Czech Republic

*e-mail: stastny@iic.cas.cz

**Chromatographic conditions**

Chromatographic analysis was carried out in a reverse phase system (RPLC-C18) on Phenomenex® Gemini column, 3 µm, C18, 110Å 100 x 4.6mm. For gradient elution, mobile phase methanol (MeOH)/water (H_2_O) acidified with formic acid (HCOOH, 0.1%) was used. The gradient was set as follows: -1.5 min → 0 min: Column equilibration (30% MeOH-HCOOH (0.1%) / 70% H_2_O-HCOOH (0.1%); 0 min → 10 min: 30% MeOH-HCOOH (0.1%) / 70% H_2_O-HCOOH (0.1%) → 90% MeOH-HCOOH (0.1%)/10% H_2_O-HCOOH (0.1%). The flow rate of the mobile phase was set to 1.0 mL·min^-1^ in the entire gradient, 25 ° C column temperature, and 10 *µL* volume injection. The measurements were performed in an ESI positive mode (ESI^+^). Data collection and evaluation was performed using the Chromeleon Chromatography Data System (CDS) software and Xcalibur program (Thermo Scientific). The HPLC method was validated with respect to linearity, recovery, precision, system suitability, selectivity, robustness, and forced degradation studies in order to prove the stability indicating the feasibility of the method. The selectivity might include degradants, matrix, etc. Decontamination studies were performed for cytostatics in order to provide an indication of the stability indicating property and the selectivity of the proposed method. The intentional decomposition was attempted at stress conditions exposing it to alkali (0.01 M NaOH) and sodium hypochlorite (5 % NaOCl) in order to evaluate the feasibility of the proposed method for the decontamination strategy of cytotoxic drugs. **Table A** shows the ionization conditions.

**Table A. ESI positive mode ionization conditions.**

| **Ionization conditions** | **ESI+** |
| --- | --- |
| Drying gas temperature [° C] | 34 |
| Evaporator temperature [° C] | 275 |
| Drying gas flow [ml/min] | 25 |
| Capillary Voltage [V] | 37 |

**HPLC method validation**

For system suitability studies, seven (*n=7*) repeated injections of stock solutions were used, and the RSD (*%*) of peak area ratio was calculated. Each solution (10 μL) was injected seven times, the areas were measured for the drug peak, and the standard deviation (*SD*) for the seven injections was calculated. Based on the obtained data, the limit of detection (*LOD*) and limit of quantification (*LOQ*) were determined using the slope (*S*) of the calibration curve and standard deviation (*SD*) of the blank sample (at a zero concentration level). The validation parameters for system suitability are shown in **Table B**. A linearity study in five concentration ranges for each cytostatic drug where the analyte response is linearly proportional to concentration was performed by preparing calibration standards in the range of 0-100 μg mL^-1^ and the analyses were performed in triplicate. The linear regression data for cytotoxic drugs showed a good linear relationship with respect to the peak area. The regression coefficient (R^2^) ranged from 0.9997 to 0.9998, indicative of an extremely significant (P < 0.0001) linear correlation.

**Table B. Validation parameters of anthracyclines analyzed by an HPLC method coupled with MS (ESI) detection.**

| **Validation parameter** | **VAL** | **PIRA** |
| --- | --- | --- |
| **Precision (% RSD)** | 5.4 | 6.4 |
| ***LOD* (μg·mL^-1^)** | 4.22 | 3.49 |
| ***LOQ* (μg·mL^-1^)** | 9.33 | 10.65 |
| **Linearity range up to (μg·mL^-1^)** | 100 | 100 |
| **Linearity (line equation and correlation coefficient, R)** | y = 1262.71 x + 2236.14  (R = 0.9998) | y = 479.10 x + 346.1  (R = 0.9997) |


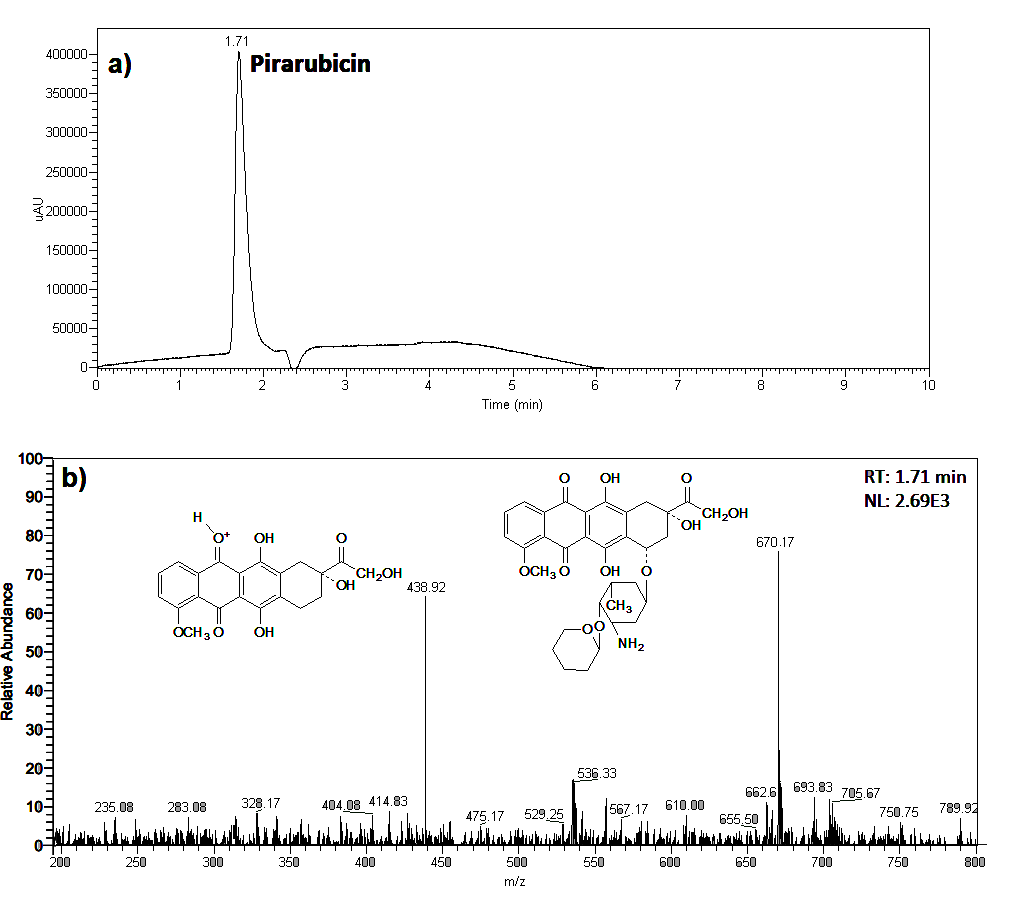


**Figure A. Extracted LC-MS chromatogram (a) and mass spectra (b) of pirarubicin (PIRA).**


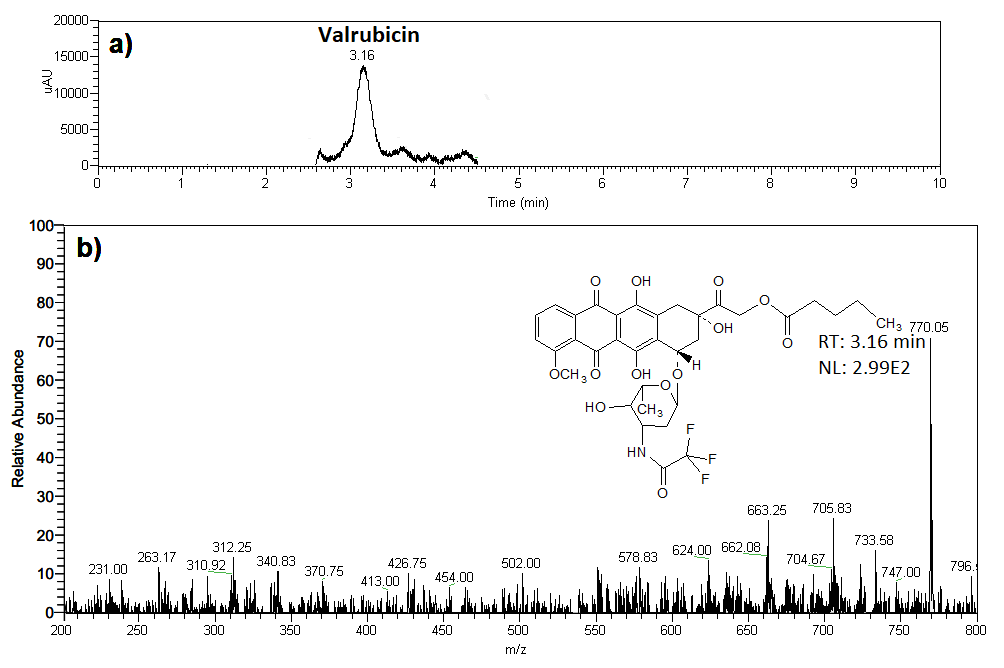


**Figure B. Extracted LC-MS chromatogram (a) and mass spectra (b) of valrubicin (VAL).**


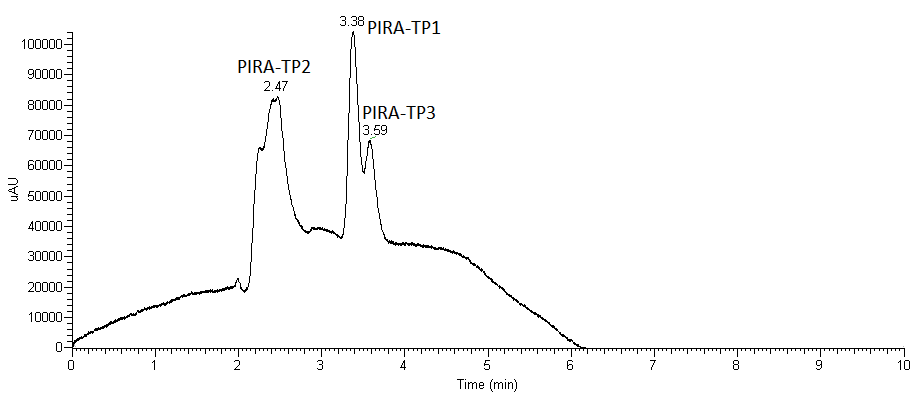


**Figure C. The typical LC-MS chromatogram of PIRA TPs obtained after the degradation in an NaClO solution (5%).**


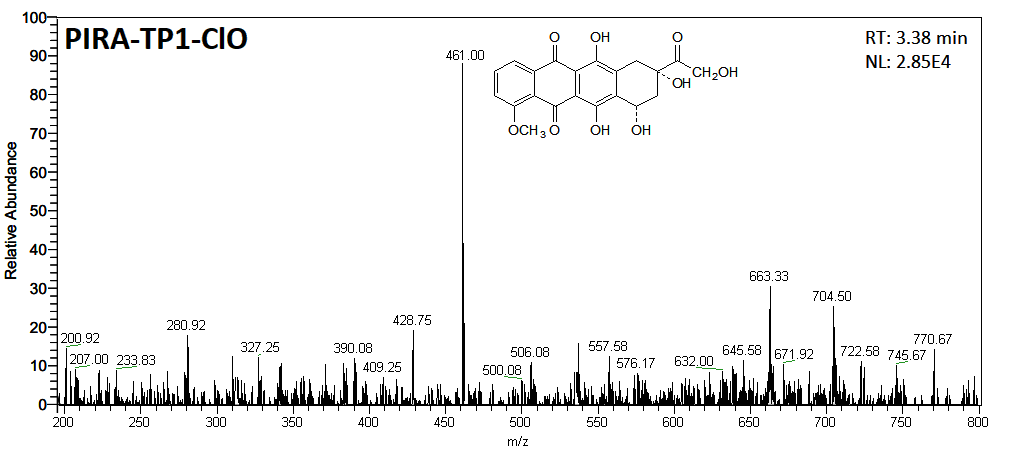

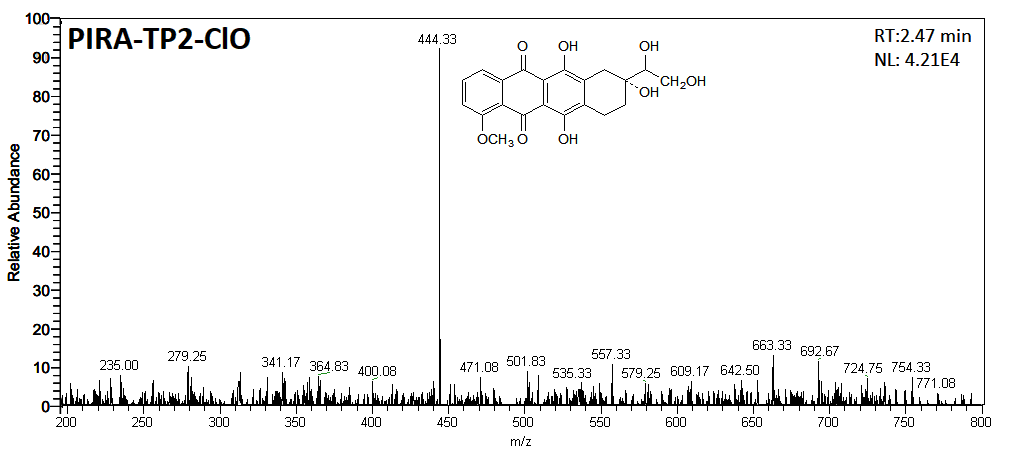

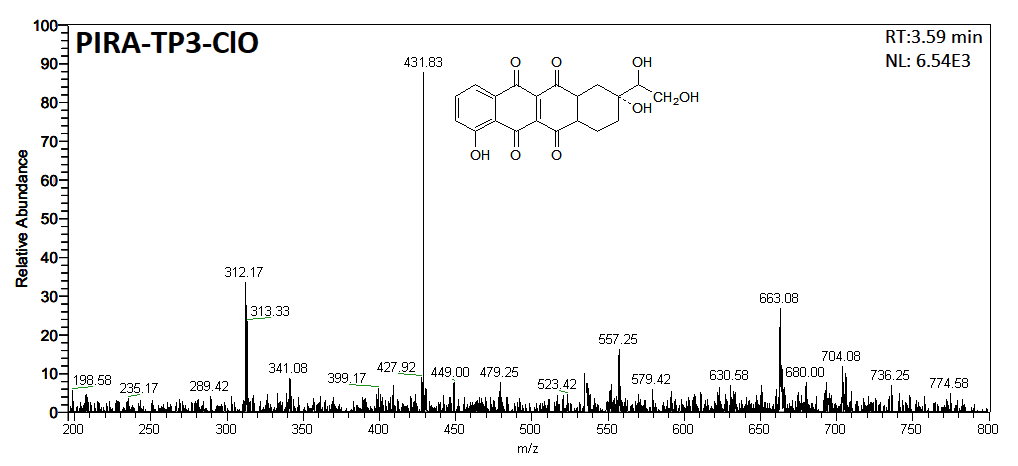


**Figure D. Mass spectra of transformation products *PIRA-TP1-OCl* (top), *PIRA-TP2-OCl* (middle) and *PIRA-TP3-OCl* (bottom) together with the proposed fragment ions.**


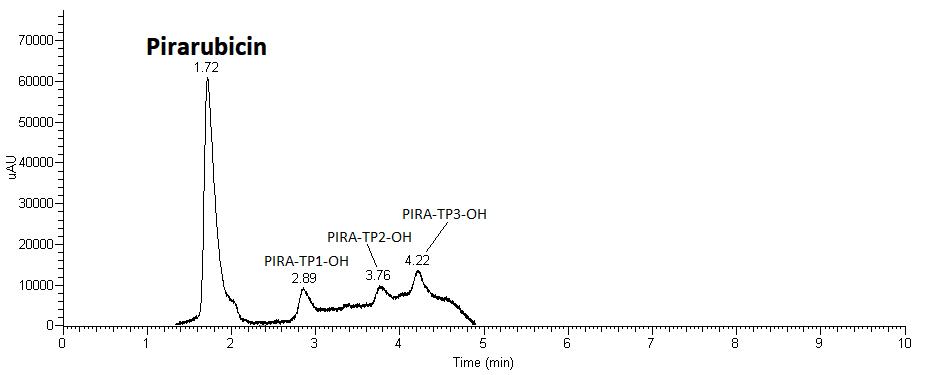


**Figure E. The typical LC-MS chromatogram of PIRA TPs obtained after the degradation in an NaOH solution (0.01 M).**


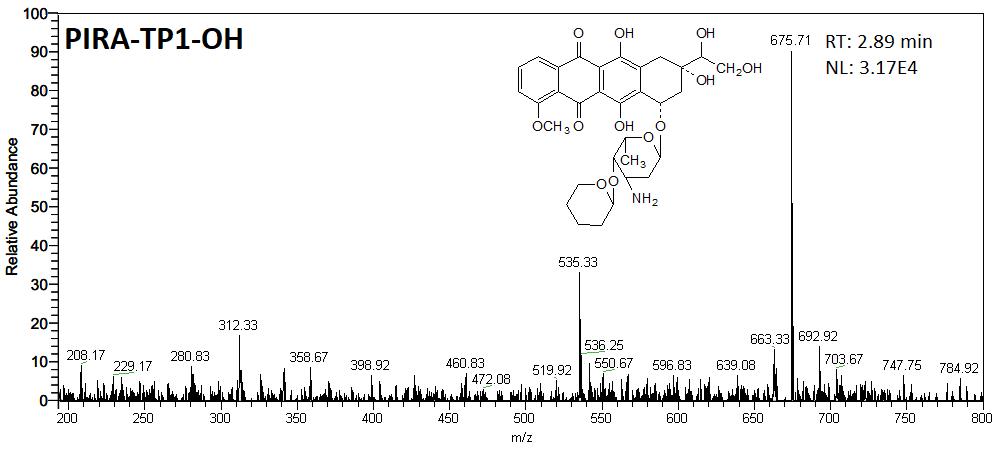

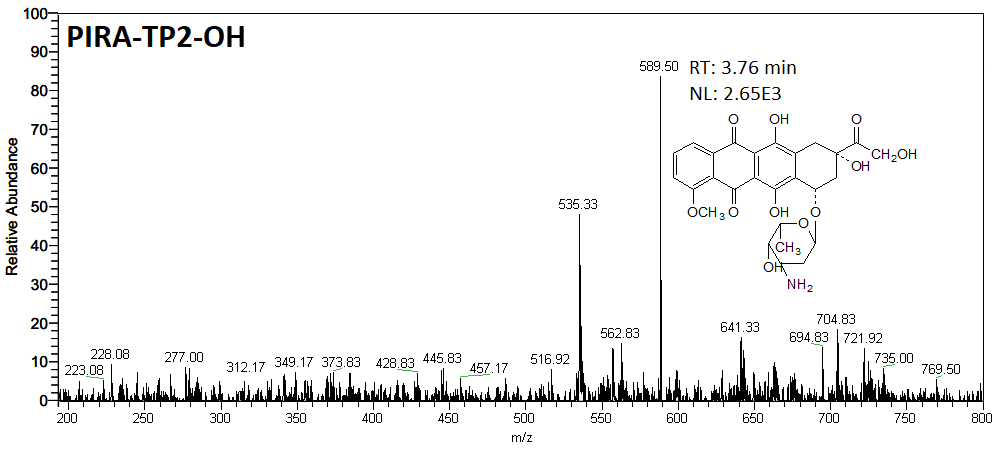

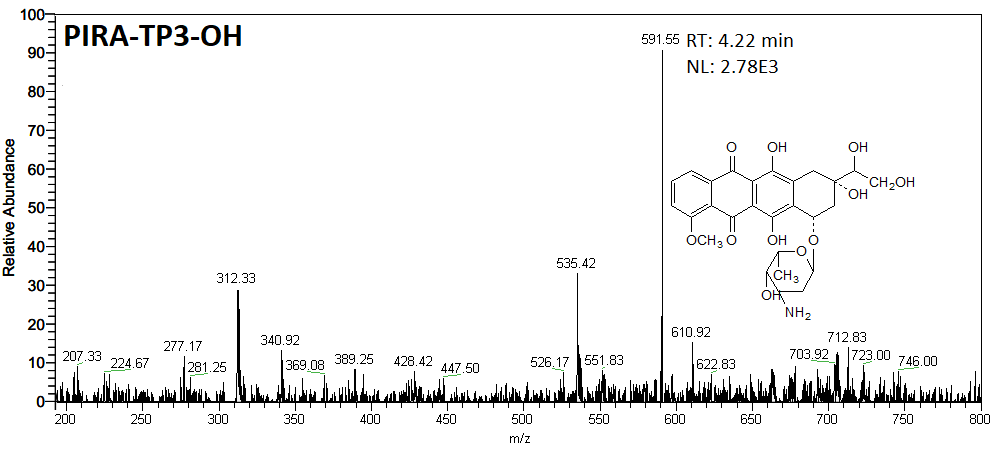


**Figure F. Mass spectra of transformation products *PIRA-TP1-OH* (top), *PIRA-TP2-OH* (middle) and *PIRA-TP3-OH* (bottom) together with the proposed fragment ions.**


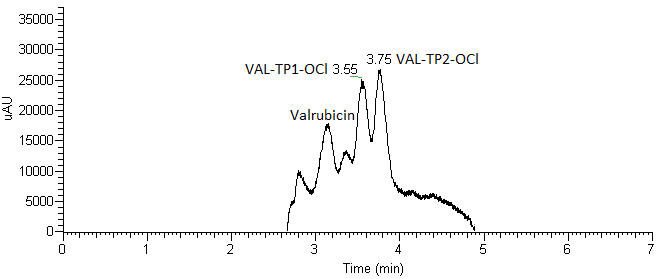


**Figure G. The typical LC-MS chromatogram of VAL TPs obtained after the degradation in an NaClO solution (5%).**


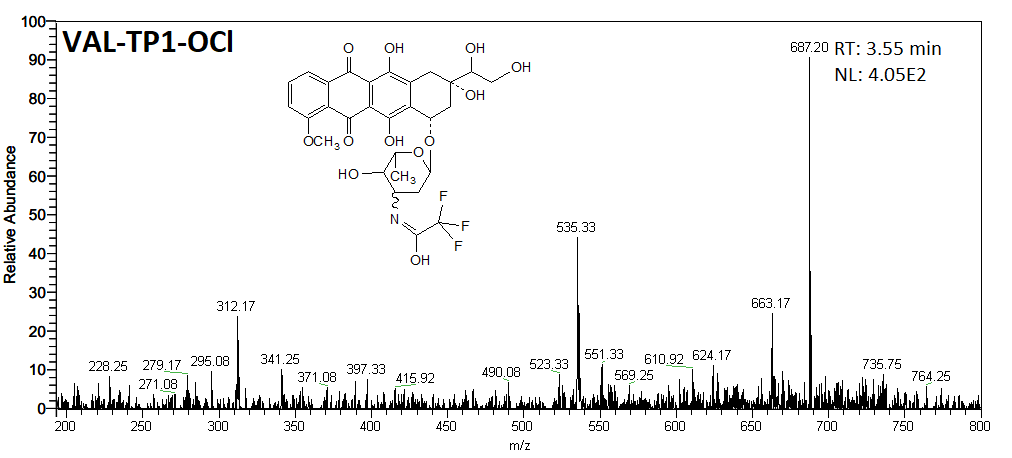

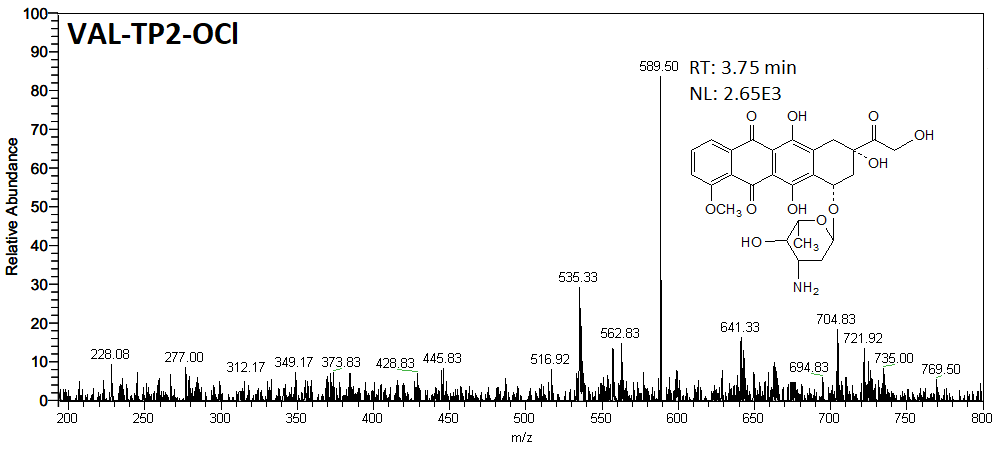


**Figure H. Mass spectra of transformation products *VAL-TP1-OCl* and *VAL-TP2-OCl* together with the proposed fragment ions.**
